# Beta-Transducin Repeats-Containing Proteins as an Anticancer Target

**DOI:** 10.3390/cancers15174248

**Published:** 2023-08-24

**Authors:** Dong Joon Kim, Yong Weon Yi, Yeon-Sun Seong

**Affiliations:** 1 Department of Microbiology, College of Medicine, Dankook University, Cheonan-si 31116, Chungcheongnam-do, Republic of Korea; kjoon95@dankook.ac.kr; 2 Multidrug-Resistant Refractory Cancer Convergence Research Center (MRCRC), Dankook University, Cheonan-si 31116, Chungcheongnam-do, Republic of Korea; 3 Department of Pathophysiology, School of Basic Medical Sciences, Academy of Medical Science, College of Medicine, Zhengzhou University, Zhengzhou 450008, China; 4 China-US (Henan) Hormel Cancer Institute, Zhengzhou 450008, China; 5 Department of Biochemistry, College of Medicine, Dankook University, Cheonan-si 31116, Chungcheongnam-do, Republic of Korea

**Keywords:** beta-transducin repeat-containing protein (β-TrCP), anticancer, cancer therapy, drug target

## Abstract

**Simple Summary:**

Beta-transducin repeat-containing proteins (β-TrCPs) are a component of the E3 ubiquitin ligase complex and function in many cellular processes to maintain protein homeostasis. Mounting evidence suggests that β-TrCPs are aberrantly upregulated in cancer tissues and are potential targets for cancer treatment. Although extensive studies have been performed to understand the mode of regulation of their substrates and its biological consequences, little attention has been paid to the mechanisms of the regulation of β-TrCPs themselves. The current review is focused on the modulation of β-TrCPs’ activities and the implications for cancer treatment.

**Abstract:**

Beta-transducin repeat-containing proteins (β-TrCPs) are E3-ubiquitin-ligase-recognizing substrates and regulate proteasomal degradation. The degradation of β-TrCPs’ substrates is tightly controlled by various external and internal signaling and confers diverse cellular processes, including cell cycle progression, apoptosis, and DNA damage response. In addition, β-TrCPs function to regulate transcriptional activity and stabilize a set of substrates by distinct mechanisms. Despite the association of β-TrCPs with tumorigenesis and tumor progression, studies on the mechanisms of the regulation of β-TrCPs’ activity have been limited. In this review, we studied publications on the regulation of β-TrCPs themselves and analyzed the knowledge gaps to understand and modulate β-TrCPs’ activity in the future.

## 1. Introduction

It is now well established that strict control of protein stability is important to maintain normal cellular physiology and prevent various diseases as well. The uncontrolled accumulation of unwanted or damaged proteins in cells or tissues leads to the disruption of homeostasis and, eventually, the development of diseases including cancers [1,2]. In addition, targeted protein degradation (TPD) utilizing the ubiquitin–proteasome system (UPS) has been acknowledged recently as a promising strategy for undruggable targets, which include the proteolysis-targeting chimeric molecule (PROTAC), small-molecule PROTAC, chloroalkane-containing PROTAC (HaloPROTAC), in-cell click-formed PROTAC (CLIPTAC), RNA-PROTAC, and transcription-factor-targeting chimera (TRAFTAC) [1,2,3,4,5,6,7,8,9,10]. The UPS is composed of a series of enzymes: E1-ubiquitin-activating, E2-ubiquitin-conjugating, and E3 ubiquitin ligases [11]. Poly-ubiquitination at the lysine 48 and/or 11 (K48/K11) residues play the role of a signal for degradation by the 26S proteasome, while mono-ubiquitination or ubiquitin chains at K6, K27, K33, and K63 are involved in various biological processes such as kinase activation, subcellular localization, DNA replication and repair, lysosomal degradation, and stress responses. More than 700 members (~5% of the human genome) of the E3 ubiquitin ligases have been identified and participate in the control of protein stability in human cells and are suggested as potential drug targets [11,12,13].

**Figure 1:**
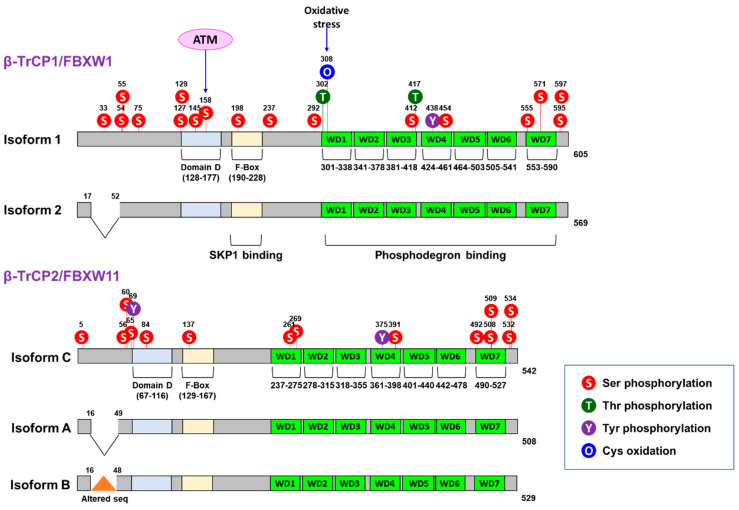
Structure of β-TrCP paralogs. Putative phosphorylation sites are predicted by NetPhos-3.1 Online Software [19,20]. ATM, ataxia telangiectasia mutated.

The beta-transducin repeat-containing protein (β-TrCP) is one of F-box proteins, which are components of the S-phase kinase-associated protein 1 (SKP1)-Cullin-1 (CUL1)-F-box (SCF)-type E3-ligase-recognizing substrates in phosphorylation-dependent ubiquitination [14,15]. Among three subfamilies of F-box proteins [16], β-TrCP belongs to the F-box with WD40 repeats (FBXW) since it interacts with its substrate through an approximately 40-amino-acid-long motif, often terminating in a tryptophan-aspartic acid (WD) dipeptide, at its C-terminus [17]. Two major paralogues of β-TrCP, including β-TrCP1 (also called FBXW1A) and β-TrCP2 (also known as FBXW11 or homologue of Slimb (HOS)), have been identified to be expressed in mammalian cells with indistinguishable biochemical properties (Figure 1) [18].

*BTRC* (the gene encoding β-TrCP1) is located at human chromosome 10q24.3 [21], which contains a gene that is mutated in prostate tumorigenesis [22] and frequently deleted in medulloblastomas [23,24]. *FBXW11* (the gene encoding β-TrCP2) is located at human chromosome 5q35.1, composed of at least 14 exons and encoding three isoforms transcribed by alternative splicing [25]. The roles of F-box proteins and their involvement in cancer have been reviewed recently [16,26]. However, the regulation of β-TrCP itself has not been well studied yet, whereas the regulation by it has been extensively investigated [27]. In this review, we analyzed the literature on the role of β-TrCP, especially in cancer and its potential as a therapeutic target for cancer treatment.

## 2. SCF^β-TrCP^ Complex and Its Substrates

β-TrCP1/2 form a homodimer or heterodimer that is mediated by N-terminal domain D before recruiting to its target protein (Figure 1) [28]. A β-TrCP dimer is a component of the SCF^β-TrCP^ complex that contains SKP1, CUL1, and RING-box protein 1/regulator of CUL1 (RBX1/ROC1) as well via direct interaction with SKP1 [29,30,31,32,33,34]. SKP1 recruits CUL1 and RBX1 to β-TrCP to form the SCF^β-TrCP^ complex [35]. In this complex, RBX1 is the E3 ubiquitin-protein ligase in association with the E2-ubiquitin-conjugating enzyme [36,37,38].

The KX_8-15_DS(P)GΨXS(P) destruction motif (where S(P) represents the phosphorylated serine residue and Ψ represents the hydrophobic residue) has been suggested as a consensus sequence for β-TrCP1 binding to its substrates such as β-catenin, inhibitor of κB (IκB)α/β/ε, and human immunodeficiency virus type-1 (HIV-1) viral protein U (Vpu) [39,40,41]. The DSGΨXS motif is phosphorylated by glycogen synthase kinase 3β (GSK3β), and the phosphorylated DS(P)GΨXS(P) motifs on the substrates play the role of signals for ubiquitinylation through β-TrCP1. Variations of the DSGΨXS motif have been found in other SCF^β-TrCP^ substrates, including the DSG(X)_4_S motif (in cell division cycle 25 homolog A (CDC25A)) [42,43] and the DSG(X)_3_S motif (in activating transcription factor 4 (ATF4) [44] and nuclear factor κ-light-chain-enhancer of activated B cells (NF-κB1) p105 [45]). Binding of β-TrCP1/2 to their substrate is dependent on the prior phosphorylation in the destruction motifs on their substrate. For example, β-TrCP1/2 only binds to p-IκBα (S32/S36), which is mediated by the IκB kinase (IKK) complex, but not non-phosphorylated IκBα [30,46,47,48].

The prior phosphorylation-independent degradation of β-TrCP substrates, such as SMA and MAD family member 3 (SMAD3), has also been reported. SMAD3 has no DSGΨXS motif [49], and its phosphorylation may not be necessary for the transforming-growth-factor-β (TGFβ)-induced degradation [49,50]. More interestingly, it has been reported that β-TrCP1 can bind to the nonphosphorylated DDG motif (DDGϕXD) in CDC25A and CDC25B, leading to the ubiquitination and degradation of CDC25A and CDC25B [51].

The HIV protein Vpu is a unique β-TrCP-binding molecule, which acts as an adaptor molecule to recruit β-TrCP1 to other cellular proteins to modulate their stability. The proteasomal degradation of cluster of differentiation 4 (CD4) is mediated by recruitment of β-TrCP1 through binding to Vpu, which contains the DSGΨXS motif [52]. Phosphoprotein Vpu acts as an adapter protein for ubiquitin-mediated degradation of CD4, which is the major cellular receptor for HIV-1. On the other hand, Vpu interferes with the degradation of IκBα, leading to the downregulation of NF-κB activity [53]. The ubiquitous casein kinase II constitutively phosphorylates DSGΨXS motifs in Vpu [54], and this p-Vpu has a dominant negative effect on β-TrCP1 function [53]. Unlike the initial demonstrations, Vpu is also degraded by β-TrCP-mediated ubiquitinylation [55].

The interaction between β-TrCP2 and β-catenin is controversial. In the yeast two hybrid system, human β-TrCP2 failed to interact with β-catenin, whereas human β-TrCP1 could interact with β-catenin [56]. On the contrary, human β-TrCP2 has been reported to bind to β-catenin in 293T cells [57]. Interestingly, the transfection of human β-TrCP2 resulted in the activation of T-cell-specific transcription-factor-1-α (TCF1α)-mediated transcription, whereas mutant β-TrCP2, lacking F-box, enhanced the TCF1α-mediated transcription in HeLa [56] or 293T [57] cells. These results suggest that some additional factor(s) lacking in yeast cells may contribute to the β-TrCP2 and β-catenin interaction.

NF-κB1 p105 is controlled by two proteolytic pathways: complete (degradation) and limited (processing to p50) [58]. Since NF-κB1 p105 also functions as an IκB, the degradation of NF-κB1 p105 results in the release of p50, v-rel avian reticuloendotheliosis viral oncogene homolog (c-REL), and v-rel avian reticuloendotheliosis viral oncogene homolog A (RELA) in the cytoplasm, and they translocate into the nucleus to modulate their target gene transcription [59,60]. NF-κB1 p105 contains a conserved motif that is similar to the DS(P)GΨXS(P) motif in the C-terminal PEST domain, and the prior phosphorylation of two serine residues by IKKα/β serves as the signal for the degradation of p105 [45,61].

In some cases, the ubiquitination of substrates results in their lysosomal degradation. Membrane receptors, such as interferon α receptor 1 (IFNAR1), are phosphorylated and subsequently bind to β-TrCP2. However, ubiquitinylated INFAR1 is degraded in the lysosome after endocytosis [62]. Endocytosis and the degradation of the growth hormone receptor (GHR) are also regulated by β-TrCP2 through GHR binding to β-TrCP2 via its ubiquitin-dependent endocytosis (UbE) motif [63]. Interestingly, β-TrCP2 binding to GHR is independent of a prior phosphorylation.

**Figure 2:**
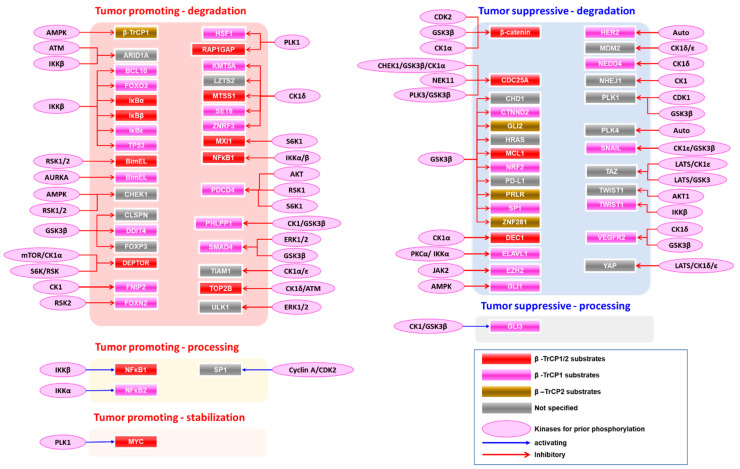
Selected targets of β-TrCPs, their priming kinases, and function in tumorigenesis.

The biological consequence of β-TrCP-mediated degradation and processing depends on the cellular context. The degradation of many substrates promotes tumorigenesis or tumor progression, while the destabilization of other substrates functions to suppress tumors (Figure 2). The detailed list of the substrates, kinases responsible for prior phosphorylation, biological consequences, and β-TrCP paralogues are summarized in Supplementary Table S1.

The Epstein–Barr virus (EBV) latent membrane protein 1 (LMP1) has been identified as a pseudo-substrate of β-TrCP2, which modulates NF-κB transcriptional activation by squelching β-TrCP2 [64]. The canonical LMP1-95-8 inhibits NF-κB activity to cause cytostatic and/or cytotoxic effects. In addition, LMP1-95-8 possesses a β-TrCP recognition-like motif, DSGHES, in its C-terminal domain [64]. Interestingly, the interaction of LMP1-95-9 with β-TrCP2 does not induce LMP1-95-9 degradation because of the lack of ubiquitin-acceptor lysines.

Similarly, HIV1 Vpu has also been reported to sequester β-TrCP1 in the cytoplasm, leading to the accumulation of its substrates including β-catenin, IκBα, and ATF4 [65]. However, the functional consequences of the inhibition of β-TrCP1 by Vpu is not fully understood.

Proto-oncogene MYC (MYC) is an important substrate of β-TrCPs [66]. Ubiquitinylation of MYC by β-TrCPs results in the stabilization of MYC rather than degradation. The formation of heterotypic polyubiquitin chains on MYC by β-TrCP antagonizes the FBXW7-mediated degradation of MYC. Of note, the downregulation of β-TrCP1 by mTORC1 inhibition is associated with a decrease in MYC levels in triple-negative breast cancer (TNBC) cells [27]. Consistent with this, mammalian target of rapamycin complex 1 (mTORC1)/P70 ribosomal protein S6 kinase (P70S6K) inhibition induces proteasomal degradation of β-TrCP, which stabilizes programmed death-ligand 1 (PD-L1) in non-small-cell lung cancer (NSCLC) cells [67].

## 3. Limited Processing by β-TrCP

A distinct β-TrCP-recognition motif is found in substrates that are processed limitedly rather than complete destruction (Table 1) [68]. In addition, a glycine-rich region (GRR) in the substrates plays the role of a STOP signal for the 26S proteasome’s digestion [69,70,71]. This limited processing by β-TrCP1 is also dependent on the prior phosphorylation of substrates [68] and subsequent neddylation [72].

Both NF-κB1 p105 and NF-κB2 p100 are limitedly processed by β-TrCP [68,73]. The limited processing of NF-κB precursors is differentially regulated by two upstream kinases, IKKβ and IKKα, respectively. Subsequently, the limited processing of NF-κB1 p105 has been reported to be independent of SCF^β-TrCP1^, whereas its degradation is mediated by SCF^β-TrCP1^, which is stimulated by IKKβ [74]. The processing of NF-κB2 p100 is primed by IKKα, which is activated by NF-κB-inducing kinase (NIK) [75]. NIK not only phosphorylates its downstream kinase IKKα, but also plays the role of a docking molecule to tether IKKα to p100.

The ubiquitous transcription factor specific protein 1 (SP1) is also regulated by proteolytic processing. Under normal conditions, SP1 is constitutively repressed by N-terminal SUMOylation [76]. Upon mitotic stimulation, SP1 is activated by proteolytic processing, which is mediated by a canonical β-TrCP-binding motif in a cyclin-A/cyclin-dependent-kinase-2 (CDK2)-mediated phosphorylation-dependent manner [77]. However, it remains to be determined which paralogue contributes to SP1 processing.

**Table 1:** β-TrCP substrates that are modified by the proteasome-mediated limited processing.

Substrate (Also Known as)	Prior Phosphorylation by	Biological Functions by β-TrCP-Mediated Processing	β-TrCP Paralogue	Role in Cancer	Ref.
GLI3	CK1/GSK3	processed into GLI3-83 repressor (GLI3R)	β-TrCP1	suppressive	[78]
NFKB1 (NF-κB p105)	IKK**β**	converted into subunit p50, leading to the formation of active NF-κB heterodimers	β-TrCP1/2	promoting	[68]
NFKB2 (NF-κB p100)	IKKα	converted into subunit p52 to form active NF-κB heterodimers	β-TrCP1	promoting	[75,79]
SP1	Cyclin A/CDK2	stabilizes and activates SP1 transcription factor	not specified	promoting	[77]

## 4. Extra Roles of β-TrCP

### 4.1. Role of β-TrCP in Regulation of Transcription

The contribution of β-TrCP1 in the transcription of genes has been reported. β-TrCP1 binds to and co-localizes with the p300 transcriptional coactivator to β-catenin target gene promoters [80]. However, unlike other β-TrCP1 binding proteins, p300 is not degraded by β-TrCP1-dependent proteolysis under normal growth conditions. In addition, β-TrCP1 also activates SMAD3-mediated transcription cooperatively with p300 [80]. The detailed molecular mechanism of the β-TrCP1-mediated transcriptional regulation remains to be determined.

### 4.2. Stabilization of Oncogene Products by β-TrCP

In contrast to the proteasomal degradation of target proteins by β-TrCPs, they can stabilize proteins through the distinct ubiquitinylation of their target proteins. The ubiquitinylation of MYC by β-TrCPs results in the stabilization of MYC rather than degradation. The formation of heterotypic polyubiquitin chains on MYC by β-TrCPs antagonizes the FBXW7-mediated degradation of MYC [81]. The stability of MYC is controlled by the SCF^FBXW7^ complex in the GSK3β-dependent phosphorylation of MYC [82,83]. However, the phosphorylation of MYC by Polo-like kinase 1 (PLK1) triggers the β-TrCP-dependent ubiquitinylation of MYC and blocks its proteasomal degradation. Consistent with this, the targeted degradation of β-TrCP by small-molecule mTORC1/P70S6K inhibitors reduces MYC protein levels in TNBC cells.

A recent study demonstrated that β-TrCP upregulates the hypoxia-inducible factor 1α (HIF-1α) protein level and its transcriptional activity by competing with its binding to heat shock protein 70 (HSP70)/the carboxy terminus of HSP70-interacting protein (CHIP), antagonizing CHIP E3 ligase activity in prostate cancer [84]. Direct binding of β-TrCP to HSP70 disrupts both HSP70-HIF-1α and HSP70-CHIP interaction. This modulation of other E3 ligases by β-TrCP is characteristic since it is not dependent on β-TrCP E3-ligase-mediated proteasomal degradation.

## 5. Association of β-TrCP with Cancer

Mounting evidence supports that β-TrCP is oncogenic [15]. The mutation or overexpression of β-TrCPs has been associated with the tumorigenesis of various cancers such as skin, gastric, prostate, and colon cancers [22,85,86,87,88]. It has been suggested that the overexpression of β-TrCP1 induces β-catenin accumulation and the activation of the downstream targets of β-catenin such as cyclin D1, glutamine synthetase, and chemotaxin 2, leading to tumorigenesis in these cancers [89]. For example, an increase in β-TrCP1 expression has been associated with colorectal cancer, which leads to the activation of β-catenin and the NF-κB pathway [87]. High levels of *BTRC* mRNA and β-TrCP1 have been found in tumor samples from patients with colorectal cancer compared to normal tissues. In addition, high β-TrCP1 levels are significantly linked to decreased apoptosis in tumor cells. In addition, the upregulation of *BTRC* mRNA and the concordant accumulation of β-TrCP1 in the cytoplasm and nucleus are found in clinical samples of patients with hepatoblastoma and hepatoblastoma cell lines [90].

Somatic *BTRC* mutations (5.3%), such as A99V, H342Y, H425Y, C206Y, and G260E, have been identified in gastric cancer samples [91]. Tumor tissues with these mutations demonstrate moderate to strong cytoplasmic accumulation of β-catenin. However, the functional consequence of these mutations remains to be determined. A 9 bp insertion or deletion (9N ins/del) polymorphism (rs16405) in the 3′-UTR of the *BTRC* gene has been negatively associated with hepatocellular carcinoma (HCC) risk in a Chinese population [92]. Among the rs16405 genotypes, the 9N ins/del and 9N del/del are associated with a reduced HCC risk compared to 9N ins/ins. In addition, the mRNA levels of *BTRC* with 9N ins/del or 9N del/del were reduced in HCC tumor tissues compared to 9N ins/ins. The 9N del disrupts the binding of *miR-920*, a negative regulator for β-TrCP1, on the 3′-UTR of the *BTRC* gene, leading to the upregulation of *BTRC* mRNA expression [92]. On the contrary, the 9N ins/del of the *BTRC* gene had no association in epithelial ovarian cancer in a Chinese population [93]. Furthermore, the cancer-related copy number variation (CNV) of the *BTRC* gene has been associated with CRC prognosis in 518 Chinese patients (amplification vs. wildtype, hazards ratio = 0.42, 95% confidence interval: 0.19, 0.97, *p* = 0.05; amplification + deletion vs. wildtype, hazards ratio = 0.39, 95% confidence interval: 0.17, 0.88, *p* = 0.023) [94].

Due to the β-TrCP1/2 control cell-cycle-dependent activity of CDK1 by regulating its upstream effectors including CDC25 [42,43], WEE1 [95], and F-box only protein 5 (FBXO5) (also known as early mitotic inhibitor 1 (EMI1)) [96,97], the dysregulation of β-TrCP1/2 may contribute to the development of tumors. The increased expression of β-TrCP1 has been reported to confer the constitutive activation of NF-κB in chemoresistant pancreatic cancer cells [98]. The targeting of β-TrCP1 by siRNA downregulates NF-κB activity and etoposide resistance in pancreatic cancer cell lines. In addition, IL-1R antagonist treatment partially inhibits β-TrCP1 expression in a chemoresistant pancreatic cancer cell line, PancTu-1. The transient expression of β-TrCP1 induces IL-1β secretion in an NF-κB-dependent manner by degrading IκBα. Consistent with this, a considerable expression of β-TrCP1 is detected in clinical samples of pancreatic ductal adenocarcinoma [98]. The overexpression of β-TrCP1 promotes cell proliferation by the activation of TNF-dependent NF-κB in diffuse large B cell lymphoma cells [99].

The potential role of β-TrCP1 in mammary gland tumorigenesis has been reported [100]. Mammary-gland-specific hypoplasia has been found in β-TrCP1^−/−^ female mice. In addition, mammary-gland-specific expression of β-TrCP1 under the control of the mouse mammary tumor virus (MMTV) long terminal repeat promoter induces the proliferation of mammary epithelia and an increased NF-κB DNA binding activity. About 40% of these mice develop tumors such as mammary, ovarian, and uterine carcinomas. On the other hand, the lymphoid-organ-specific expression of β-TrCP1 by the CD4 promoter displays no effects on these organs.

The low-level expression of β-TrCPs has been reported in glioma tissue [21] and associated with the poor survival of patients with glioma [101]. A subsequent study demonstrated that the overexpression of β-TrCP reduces migration, invasion, and proliferation in glioma cell lines [102].

The loss of β-TrCP1 is also found in several lung cancer cell lines and subsets of lung cancer specimens [103]. In such cases, the stable expression of β-TrCP1, potentially through the downregulation of CDC25A, leads to the negative regulation of cell motility, cell growth in soft agar, and tumor growth in xenografts.

A high level of expression of β-TrCP2 has been reported in human cancer cell lines and primary breast tumors [104]. On the contrary, the downregulation of β-TrCP2 has been reported in clinical chondrosarcoma samples [105]. In addition, the recovery of β-TrCP2 suppresses chondrosarcoma cell growth and induces apoptosis. A high level of expression of β-TrCP2 has also been reported in patients with lymphocytic leukemia [106]. The overexpression of β-TrCP2 in lymphocytic leukemia cells promotes cell proliferation in vitro and tumor formation in vivo through the stimulation of cell cycle progression.

The tumor suppressive function of β-TrCPs is occasionally impaired by the stabilization of their substrates through decreased phosphorylation and/or binding capability. The oncogenic activation of β-catenin is achieved by the decreased phosphorylation of the degradation motif (degron) in cancer cells [107]. Interestingly, the induction of β-TrCPs has been reported in cells expressing an oncogenic β-catenin mutant, which leads to the activation of the NF-κB transcription factor [88]. In addition, the epigenetic regulation of *BTRC* and *AXIN2* by promoter hypermethylation and histone deacetylation has been associated with nuclear β-catenin accumulation in NCSLC cell lines and patient samples [108]. The stabilization of the prolactin receptor (PRLR) is also correlated with enhanced expression of it in breast cancer. The reduced phosphorylation of PRLR in phospho-degron results in inefficient recruitment of β-TrCP and the accumulation of PRLR in breast cancer cells and tissues [109]. Targeting PRLR has been reported to exert anticancer effects on breast cancer cells both in vitro and in xenograft models [110]. In human medulloblastomas and neuroblastomas, the RE1-silencing transcription factor (REST) plays the role of an oncogene and evades β-TrCP1-mediated degradation by C-terminal truncations [18]. The inactivation of kinases for prior phosphorylation is another mechanism of evasion of β-TrCP-mediated degradation in cancer. The inactivation of GSK3β, the kinase for CDC25A priming, has been associated with CDC25A overproduction in human tumor tissues [111]. The stabilization of PRLR is also mediated by the human epidermal growth factor receptor 2 (HER2)-/RAS-signaling-induced inhibitory phosphorylation of GSK3β in breast cancer cells, and elevated PRLR levels are correlated with GSK3β inactivation in breast cancer specimens [112].

Taken together, β-TrCP1/2 may function either as an oncogene or tumor suppressor in a cellular-context-dependent manner. A detailed understanding of the complex regulation of β-TrCP1/2 in the differential cellular context will provide new insights into tumorigenesis or tumor suppression and an alternative strategy to develop novel targeted therapeutics.

## 6. Regulation of β-TrCP Activity

### 6.1. Upstream Effectors of β-TrCP Activity

In general, the endogenous levels of β-TrCPs are known to be low, whereas the upregulation of β-TrCPs is often found in cancer cell lines and primary tumors [17,22,85,86,87,88,113,114]. However, the regulation of β-TrCPs’ mRNA transcription, protein stability, and subcellular localization remains largely unknown [17]. For example, β-TrCP1/2 contain multiple putative phosphorylation sites (Figure 1). However, protein kinases responsible for their phosphorylation have not been elucidated yet, although several protein kinases have been reported to modulate β-TrCP activity (Table 2). Currently, ATM is the only protein kinase that has been identified to phosphorylate the S158 of β-TrCP1 (Figure 1), leading to enhanced β-TrCP1 activity toward the proteasomal degradation of β-TrCP2 [115].

It has been reported that the expression of *BTRC* (the gene encoding β-TrCP1) mRNA and β-TrCP1, but not *BTRC* gene transcription, is elevated by WNT/β-catenin/transcription factor 1 (TCF1) signaling, forming a negative feedback loop to control the WNT/β-catenin/TCF1 pathway [88]. GSK3 activity is blocked by the expression of WNT1/2 or constitutively active mutant AKT-upregulated *BTRC* mRNA, but not *FBXW11* (the gene encoding β-TrCP2) mRNA, via a TCF1-mediated transcription-dependent manner. The upregulation of *BTRC* mRNA results in an abundance of β-TrCP1. In addition, constitutively active AKT may contribute to the degradation of IκB through the upregulation of β-TrCP1 in cancer cells [88]. A protein insulin-like growth factor 2 mRNA-binding protein 1 (IGF2BP1) (also called coding region determinant-binding protein (CRD-BP)) binds to and stabilizes *BTRC* mRNA, leading to an increase in the β-TrCP1 level both in cells and in vivo [66]. IGF2BP1 is a transcription target of β-catenin. IGF2BP1 also binds to and stabilizes *MYC* mRNA and upregulates the MYC protein in colorectal cancer [66]. A high level of expression of IGF2BP1 and β-TrCP1 has been associated with nuclear accumulation of β-catenin in colorectal tumor samples. Interestingly, β-TrCP1/2 stabilizes MYC by antagonizing FBW7-dependent degradation [81]. Consistent with this, β-catenin/TCF1 signaling induces IGF2BP1 expression. In addition, the CUL1-SKP1-FBXW8-CUL7 complex promotes cell migration by activating β-catenin via directing the proteasomal degradation of β-TrCP1 [126]. FBXW8 interacts with β-TrCP1 and induces the proteasomal degradation of β-catenin, leading to CDC25A-mediated cell cycle transition from the G1-phase to the S-phase in a mitogen-activated protein kinase (MAPK) pathway-dependent manner [127].

GSK3 modulation by Wnt and β-catenin stabilization remains a question. β-TrCP ubiquitinates GSK3β, and focal adhesion kinase (FAK) and proline-rich tyrosine kinase 2 (PYK2) enhance the Wnt/β-catenin pathway through ubiquitinated GSK3β phosphorylation at Y216. The ubiquitination and phosphorylation of the GSK3β/β-TrCP complex reinforces the Wnt signaling pathway and β-catenin stabilization, resulting in promoting intestinal tumorigenesis [163]. However, β-catenin stability and the function of the GSK3β/β-TrCP complex remain to be elucidated.

Interestingly, contrary to β-TrCP1, the activation of the WNT/β-catenin pathway downregulates β-TrCP2 transcription in HEK293T and probably in colorectal tumors [113]. In cells lacking β-TrCP1 expression, but expressing β-TrCP2, the activation of WNT/β-catenin downregulates β-TrCP2 expression, leading to the amplification of β-catenin signaling and the inhibition of NF-κB activity.

Constitutively active NF-κB expression has been reported in human melanoma cells [164]. Oncogenic mutant BRAF^V600E^ induces β-TrCP2 expression and concordantly activates IKK activity to induce the degradation of IκBα in mouse melanocytes [119]. Consistent with this, the knockdown of BRAF^V600E^ decreases *FBXW11* promoter activity and β-TrCP2 expression in human melanoma cells [119]. The pharmacological inhibition of RAF or MAPK/extracellular-signal-regulated kinase 1 (MEK1) downregulates β-TrCP2 expression in human melanoma cells. In addition, mitogens such as 12-*O*-tetradecanoylphorbol-13-acetate (TPA) induce *FBXW11* mRNA expression through MAPK pathway activation in a normal fibroblast cell line, NIH/3T3 [104].

Post-translational modification of β-TrCPs remains largely unknown [27]. As shown in Figure 1, many putative phosphorylation residues are found in β-TrCPs. However, the corresponding kinases are waiting to be discovered. Previously, mTORC2, but not mTORC1, has been reported to inhibit β-TrCP degradation in TNBC cells [27]. The pharmacological inhibition of mTORC2 by a small-molecule inhibitor, WYE-354, induces the reduction of β-TrCP levels in a dose-dependent manner. On the contrary, rapamycin does not. In addition, treatment by a PI3K/mTOR inhibitor, PI-103, reduces the serine/threonine phosphorylation of β-TrCP and reduces its protein levels. The PI-103-induced degradation of β-TrCP is dependent on proteasomal activity since MG132 treatment abolishes its degradation in the presence of PI-103. In addition, the knockdown of β-TrCP1 by siRNA markedly reduces the proliferation of TNBC cells in vitro [27]. Taken together, the phosphorylation of β-TrCP may contribute to its stability in cancer cells.

Both β-TrCP paralogs are functionally redundant due to their lack of selectivity in substrate recognition. In addition, β-TrCP paralogs reciprocally regulate each other. AMPK is activated and phosphorylates β-TrCP1 for subsequent SCFβ-TrCP2-mediated ubiquitination and degradation in glucose deprivation or serum starvation, and SCFβ-TrCP1 promotes β-TrCP2 ubiquitination and degradation mediated by some unknown kinase(s) [116]. In addition, β-TrCP2 inhibits autophagy and senescence and promotes cell proliferation and migration, whereas β-TrCP1 suppresses cell growth in TNBC cells. In addition, β-TrCP2, not β-TrCP1, governs the activity of mTORC1, a central regulator of autophagy and growth, by preferentially degrading the DEP domain-containing mTOR-interacting protein (DEPTOR) and regulated in development and DNA damage response (1REDD1). DEPTOR and REDD1 are two well-known substrates of SCF^β-TrCP^ and inhibitors of mTORC1. Thus, βTrCP2 acts as a dominant paralog with oncogenic properties in the regulation of cell autophagy and growth [116]. Interestingly, it has been reported that the β-TrCP-mediated degradation of HER2 in HER2+ breast cancer cells was abrogated by DEPTOR through its interaction with HER2 [165]. DNA damage also activates β-TrCP1 via phosphorylation at S158 by ATM [115]. ATM-mediated phosphorylation protects β-TrCP1 from β-TrCP2-mediated degradation. Phosphorylated β-TrCP1 enhances the proteasomal degradation of β-TrCP2 and mouse double minute 2 (MDM2), leading to G2/M cell cycle arrest to promote DNA repair in response to DNA damage. Degradation of MDM2 is mediated by the inhibition of the polyubiquitination at K63 of MDM2 by β-TrCP2, which is directly ubiquitinated at K48 by β-TrCP1 and subsequently undergoes proteasomal degradation [115]. β-TrCP1 regulates MDM2 negatively by abrogating the K63-linked ubiquitination of MDM2 by β-TrCP2 and promoting the polyubiquitination of MDM2 at K48. Of note, the polyubiquitination of TNF receptor-associated factor 6 (TRAF6) at K63 is reduced by β-TrCP to inhibit lipopolysaccharide (LPS)-induced IKK activation [166]. These results imply that β-TrCP paralogs play the role of a differential cellular process by the reciprocal regulation of each other upon various extracellular and intracellular signals. Since β-TrCP1 and β-TrCP2 form either a homodimer or a heterodimer with differential potency in promoting substrate degradation [28], further studies are needed to understand the fine regulation of cellular processes by β-TrCP paralogs.

The overexpression of the tumor suppressor RAS-associated domain-containing protein 1A (RASSF1A) shows an antiproliferative effect and decreasing cyclin D1 levels, potentially by restricting cells in the retinoblastoma (RB) cell cycle checkpoint to prevent them entering the S-phase [167]. In addition, RASSF1A inhibits SCF^β-TrCP^ activity to allow the G-to-S transition through the upregulation of the levels of FBXO5/EMI, which blocks anaphase-promoting complex (APC) activity [130]. The underlying mechanism of RASSF1A-mediated inhibition of SCF^β-TrCP^ activity remains elusive.

RASSF1C, an isoform of tumor suppressor RASSF1, has been reported to inhibit β-catenin degradation through direct interaction with β-TrCP1 [131]. The interaction between RASSF1C and β-TrCP1 is mediated by the SSGYXS motif in the N-terminus of RASSF1C, which is absent in RASSF1A. Although the SSGYXS motif is reminiscent of the phospho-degron motif that is recognized by β-TrCP1, RASSF1C binding to β-TrCP1 is not mediated by WD40 repeats in β-TrCP1. The binding of RASSF1C to β-TrCP1 may inhibit the interaction of β-catenin with β-TrCP1, leading to a change in the β-catenin subcellular localization from the nucleus to the cytoplasm. Interestingly, the silencing of RASSF1A in cells expressing both RASSF1A and RASSF1C is enough to induce β-catenin accumulation. RASSF1C is suggested to be a pseudosubstrate or negative modulator of β-TrCP1 to block the degradation of β-TrCP1 substrates [131].

The inhibition of Janus Kinase 2 (JAK2) either by small-molecule inhibitor AG490 or by knockdown with shRNA results in an increase in β-TrCP and GSK3α/β at both the mRNA and protein level in both human leukemia Jurkat cells and human erythroleukemia HEL cells [168]. JAK2-blockade-induced β-TrCP activation leads to the degradation of IκB and the nuclear translocation of NF-κB. However, the exact molecular mechanism of JAK2-reguated β-TrCP activity remains to be determined.

Proto-oncogene SRC (SRC) is a nonreceptor tyrosine kinase that inhibits the Hippo pathway from enhancing tafazzin (TAZ) decay mediated by β-TrCP. TAZ is a transcription coactivator, shuttling from the cytoplasm to the nucleus. Hippo pathway kinase large tumor suppressor homolog 1/2 (LATS1/2) reduces TAZ nuclear localization and minimizes TAZ cytoplasmic levels by the E3 ligase β-TrCP [169]. The polyomavirus-middle-T-antigen (PyMT)-mediated SRC activation inhibits TAZ degradation via β-TrCP, leading to the expression of the *CTGF* and *ANKRD1* genes, which are nuclear targets of TAZ and the YES-associated protein (YAP). The inhibition of β-TrCP by SRC is also observed with IκB [138]. However, the mechanism involved in the attenuation of β-TrCP E3 ubiquitin ligase activity by SRC remains to be determined. The stability of TAZ in chondrocytes is negatively regulated by tumor protein 53 (TP53) by the physical interaction between TP53 and TAZ, promoting TAZ degradation by β-TrCP [170]. A β-TrCP substrate, TIAM1, also contributes to TAZ degradation by enhancing β-TrCP–TAZ interactions to inhibit the invasion of intestinal epithelial cells [171].

Centromere protein W (CENP-W) and heterogeneous nuclear ribonucleoprotein U (hnRNP U) interact with the region of F box and the WD40 domain of β-TrCP1, respectively [122]. The interaction complex leads to a stable shuttling complex, resulting in the accumulation of β-TrCP1 in the nucleus and promoting cell migration. It has been proposed that CENP-W may enhance the oncogenic potential of β-TrCP1 by promoting its nuclear accumulation [122].

Ubiquitin-specific peptidase (24USP24) belongs to the superfamily of deubiquitinases (DUBs), which have been correlated with cancer progression. Elevated USP24 in malignant cancer cells and M2 macrophages promotes metastasis by positively regulating IL-6 expression through stabilizing p300 and β-TrCP, leading to increases of histone-3 acetylation and NF-κB and decreases in DNA methyltransferase 1 (DNMT1) and IκB levels [142]. However, the underlying mechanism of the USP24-mediated stabilization of β-TrCP remains to be elucidated.

USP47 is a member of the ubiquitin-specific proteases (USPs), which regulate the activity of E3 ubiquitin ligases. USP47 protein levels are little changed, although USP47 binds specifically to β-TrCP1/2 at the WD-repeat domain [143]. Endoplasmic reticulum aminopeptidase 1 (ERAP1) interacts with USP47 tumorigenesis via β-TrCP degradation by competing with USP47-β-TrCP association [172]. Eventually, the inhibition of USP47 stabilization by ERAP1 induces GLI transcription factors’, the final effectors of the Hh pathway, activation. Knockdown of USP47 induces CDC25A accumulation and the inhibition of tumor growth. Interestingly, β-TrCP directly binds to the USP47 motif at DSGXXXS and regulates USP47 ubiquitination [173]. In addition, USP47 deubiquitinates itself and positively regulates β-catenin stabilization [173]. Recently, endoplasmic reticulum aminopeptidase 1 (ERAP1) has been reported to interact with USP47, promoting tumorigenesis via β-TrCP degradation [172]. ERAP1-USP47 binding disrupts the USP47-β-TrCP binding and subsequent ubiquitination and proteasomal degradation of β-TrCP, leading to increases in the levels of the GLI1 and GLI2 proteins in the Hedgehog (Hh) pathway. These results suggest that β-TrCP is a potential target to treat Hh-driven cancers.

RING-box protein 2 (RBX2) (also known as sensitive to apoptosis gene (SAG)) regulates β-TrCP1’s half-life by facilitating the formation of the K11-linked ubiquitinylation chain on β-TrCP1 [132]. Since RBX2 is a bona fide anti-apoptotic protein [174], the degradation of β-TrCP1 by RBX2 may play a role in tumorigenesis.

Previously, it has been demonstrated that extracellular stresses, such as ultraviolet (UV) radiation, hydrogen peroxide (H_2_O_2_), and tumor necrosis factor α (TNFα), upregulate β-TrCP1 via the elevation of its mRNA level in 293T cells [114]. The stabilization of β-TrCP1 by H_2_O_2_ is achieved by the oxidative modification of cysteine 308 residues in β-TrCP1 [175]. Since C308 is required for maximal binding between β-TrCP1s, the oxidation of cysteine thiols results in the diminished degradation of IκBα in lipopolysaccharide-stimulated cells in response to H_2_O_2_ exposure, leading to the anti-inflammatory effects of H_2_O_2_ in immune cells such as neutrophils. In addition, c-JUN N-terminal kinase (JNK) and p38 have also been reported to upregulate β-TrCP1 through the stabilization of *BTRC* mRNA [114]. Constitutively active mutant upstream kinases of JNK, such as JNK kinase 2 (JNKK2) or MAPK kinase 6 (MKK6), also induce *BTRC* mRNA. On the contrary, MEK1 or IKKβ does not induce β-TrCP1 accumulation. Again, the effector molecules for JNK/p38, which mediate *BTRC* mRNA stabilization, are largely unknown.

The upregulation of Tetraspanin 15 (TSPAN15) has been reported in esophageal squamous cell carcinoma (OSCC) tissues [141]. In OSCC cells, TSPAN15 binds to β-TrCP1 to enhance the proteasomal degradation of p-IκBα, leading to the activation of NF-κB transcription. The induction of NF-κB target genes, such as ICAM1, VCAM1, uPA, MMP9, TNFα, and CCL2, promotes the metastatic capabilities of OSCC cells.

Mammalian miRNAs regulate various genes’ expression through binding on the 3′-UTR of target mRNAs, leading to mRNA degradation. A series of miRNAs has been reported to downregulate either *BTRC* or *FBXW11* mRNA (Table 2). For example, *BTRC* and *FBXW11* contain a highly conserved miR-10a [150] and miR-182 [153] binding site, respectively, within their 3′-UTRs. miR-10a and miR-182 directly bind to the 3′-UTR of *BTRC*, and *FBXW11* degrades their mRNAs in human aortic endothelial cells [150] and in pancreatic cancer cells [153], respectively. The overexpression of miR-182 has been reported to promote pancreatic cancer cell proliferation and migration in a β-TrCP2-dependent manner [153].

Long non-coding RNAs (lncRNAs) have a crucial role in the signaling cascade of tumorigenesis and chemoresistance. LncRNAs also contribute to the modulation of β-TrCP activity (Table 2). LncRNA *SLC7A11-AS1* expression is elevated in gemcitabine-resistant pancreatic ductal adenocarcinoma (PDAC) cells [161]. *SLC7A11-AS1* interacts with the F-box motif of β-TrCP1, preventing NRF2 ubiquitination and subsequent proteasomal degradation in the nucleus. Stabilized NRF2 reduces intracellular ROS for the maintenance of PDAC stemness and chemoresistance [161].

Circular RNA has been reported to regulate β-TrCP activity. *CircHIPK3* functions as a scaffold for ELAV-like protein 1 (ELAVL1) and β-TrCP1 to enhance β-TrCP1-mediated ubiquitination and degradation of ELAVL1, leading to a decrease in the p21 level and cardiac senescence with a concordant increase in telomere length [145]. Studies suggest that *circHIPK3* has a dual role in tumorigenesis and tumor progression [176,177,178]. Further studies are needed to delineate the *circHIPK3*-mediated regulation of β-TrCP1 activity over a diverse set of its substrates and their functional consequences in tumor development.

### 6.2. Modulation of β-TrCP Activity by Protein–Protein Interactions

Protein–protein interactions function as regulators of β-TrCP activity or substrate binding (Table 3). Competitive inhibition of the interaction between β-TrCPs and their substrates controls β-TrCPs’ function. For example, 14-3-3ζ (also known as YWHAZ) competitively binds to β-catenin to dissociate it from β-TrCP binding [179]. Since 14-3-3ζ has been reported to be elevated in many human cancers including NSCLC, the 14-3-3ζ-mediated upregulation of β-catenin by increasing its stability could be a mechanistic basis for lung cancer malignancy [179].

Interestingly, transcription factors are involved in the regulation of β-TrCP activity through physical interactions. Activating enhancer-binding protein 2-β (AP2-β) suppresses the proliferation of cervical cancer cells [180]. AP2-β binds to β-TrCP and enhances its activity toward β-catenin. A negative correlation between AP2-β and the β-catenin protein is found in clinical cervical cancer tissues. A xenograft study further demonstrated that AP2-β reduces cervical tumor growth by inhibiting the expression of WNT target genes.

Estrogen receptor α (ERα) is a nuclear hormone receptor that is specifically activated by 17β-estradiol (E2) [188]. The treatment by E2 of HA22T HCC cells results in enhanced binding of ERα to β-catenin, triggers the binding of β-catenin and β-TrCP, and promotes the degradation of β-catenin, leading to the inhibition of the migration and invasion of HA22T cells [181]. In addition, transcription factor AP2-β also activates the CK1/GSK3β-mediated phosphorylation-dependent proteasomal degradation of β-catenin by binding to β-catenin and β-TrCP, leading to the suppression of cervical cancer cell proliferation [180].

RASSF5 is a tumor suppressor and direct RAS effector [189]. RAS can stimulate SCF^β-TrCP1^ via RASSF5. Activated RASSF5 directly forms a complex with β-TrCP1 and enhances β-catenin degradation [182]. Interestingly, RASSF5 does not affect IκB stability. The mechanism of this differential regulation of β-TrCP1-mediated degradation by RASSF5 remains to be determined.

Tribbles homolog 2 (TRIB2), a substrate of β-TrCP1 [190], reciprocally inhibits β-TrCP1 activity by protein–protein interaction to stabilize YAP in liver cancer cells [183]. In addition, TRIB2 contributes to the negative regulation of WNT/β-catenin/TCF4 signaling specifically in liver cancer cells by physically binding to β-TrCP, COP1, and SMAD ubiquitination regulatory factor 1 (SMURF1) [191]. In addition, SMURF1 increases the protein stability of β-TrCP by reducing the autoubiquitination of β-TrCP in liver cancer cells [139]. Mechanistically, TRIB2 enhances nuclear co-accumulation of β-TrCP E3 ligases and β-catenin, promoting the destabilization of β-catenin and TCF4 in liver cancer. Taken together, the TRIB2–β-TrCP interaction may contribute to the tight control of β-TrCP activity in a spaciotemporal and/or tissue-specific manner in normal physiological conditions, and the dysregulation of this protein–protein interaction functions in tumorigenesis. Interestingly, TRIB3 has also been reported to modulate β-TrCP activity through direct binding to TAZ. Taken together, WNT signaling is finely regulated by β-TrCP at multiple levels.

Tripartite motif-containing proteins (TRIMs) such as TRIM9 and TRIM67 also modulate β-TrCP by direct physical interaction [185,186]. The binding of TRIM9 or TRIM67 prevents β-TrCP from binding to its substrates and stabilizing IκBα, leading to the inhibition of NF-κB activation [123,192].

Ubiquitin-domain-containing protein 1 (UBTD1) interacts with the E2-ubiquitin-conjugating enzymes of the ubiquitin proteasome system [193,194]. UBTD1 interacts with the YAP degradation complex and enhances β-TrCP-dependent YAP degradation [187]. The mechano-transducer C-X-C chemokine receptor type 4 (CXCR4) downregulates UBTD1 and stabilizes YAP in HCC cells in response to the extracellular matrix’s stiffness [187]. The stability and activation of YAP1 are also regulated by apurinic/apyrimidinic endonuclease 1 (APE1), which binds to β-TrCP, possibly competing with the YAP1–β-TrCP interaction in response to acidic bile salt exposure in esophageal adenocarcinoma cells [195]. Interestingly, extracellular matrix stiffness induces the degradation of mammalian STE20-like protein kinase 2 (MST2), a Hippo kinase, by β-TrCP, in human breast epithelial cells [196]. Enhanced MST2 degradation in human breast epithelial cells is also induced by the hyperactivation of integrins via intergrin-linked kinase (ILK) [196]. It remains to be determined whether there is a differential regulation of the Hippo pathway via β-TrCP in normal and cancerous cells in response to extracellular matrix stiffness or not. MEK1 also interacts with YAP to promote its stability independent of MST/LATS/Hippo and ERK in liver cancer cells [197]. Importantly, MEK1–YAP interaction promotes tumorigenesis in liver cancer cells.

In addition, blocking β-TrCP-mediated proteolysis by additional effector molecules has also been reported. In cancer cell lines and metastatic tumors, the upregulation of SNAIL has been associated with NF-κB activation [192]. Interestingly, NF-κB induces COP9 signalsome 2 (CSN2), which blocks the β-TrCP-mediated degradation of SNAIL by inhibiting the interaction of SNAIL with β-TrCP and GSK3β, leading to cell migration and invasion in response to the inflammatory cytokine TNFα [192]. Epidermal-growth-factor (EGF)-induced extracellular signal-regulated kinase 2 (ERK2) activation phosphorylates CSN6 at S148, leading to β-catenin stabilization through blocking β-TrCP and colorectal cancer development [123].

### 6.3. Modulation of β-TrCP Activity by Viral Proteins

Interestingly, viral oncoproteins target β-TrCP to suppress immune reaction or tumor suppression (Table 4). The A49 protein of poxvirus inhibits β-TrCP-dependent IκBα degradation by molecular mimicry [198]. It contains a motif conserved in IκBα and phosphorylated by IKKβ and subsequently binds to β-TrCP to prevent IκBα ubiquitination and degradation. As a result, the activity of NF-κB is reduced and immune evasion is promoted. The adenoviral E1A protein upregulates β-TrCP1 by unknown mechanisms to induce β-TrCP1-dependent degradation of the REST tumor suppressor, leading to viral transformation [199].

A study with viral protein revealed the cullin 3 (CUL3)–RBX1 ubiquitin ligase complex as a β-TrCP1 E3 ligase [200]. Nonstructural protein 1 (NSP1) from several human rotaviruses also negatively controls NF-κB signaling by inducing β-TrCP degradation [201,202]. NSP1 phosphorylation by CKII recruits β-TrCP and promotes β-TrCP degradation [203]. A sequence motif similar to the β-TrCP-recognition motif of IκB is found in the C-terminal region of NSP1. Rotavirus NSP1 mediates the CUL3–β-TrCP1 interaction in the Golgi apparatus and subsequently induces β-TrCP1 degradation in a proteasome-dependent manner.

The ORF61 of simian varicella virus and the varicella-zoster virus also inhibit the NF-κB pathway by binding to β-TrCP [204]. The ORF2 of the hepatitis E virus binds to β-TrCP to inhibit IκBα degradation, leading to the suppression of host immune reaction [205].

**Table 4:** Viral effectors regulating β-TrCP activity.

Viral Effector	Virus	β-TrCP Isoform	Biological Consequences	Role in Cancer	Ref.
A49	poxvirus	not specified	inhibits NF-κB activity by preventing the degradation of IκBα	suppressive	[198]
E1A-5/E1A-12	adenovirus	not specified	upregulates β-TrCP, leading to degradation of the REST tumor suppressor	promoting	[199]
E17	human papilloma virus 16	not specified	reduces DNA damage checkpoint responses and promotes mitotic progression by downregulating cullin 1, β-TrCP, AURKA, and PLK1	promoting	[206]
*EBV-miR-BART10-3p*	Ebstein–Barr virus	β-TrCP1	binds to the coding region of *BTRC* mRNA and induces its stability, leading to the facilitation of EMT and the promotion of the metastasis of nasopharyngeal carcinoma	promoting	[207]
NS1	influenza A virus	β-TrCP1	induces β-TrCP1 proteasomal degradation through physical interaction with β-TrCP1, leading to elevation of IκBα	-	[208]
NSP1	rotavirus	not specified	decreases the level of β-TrCPs	promoting	[201]
ORF6	simian varicella virus and varicella-zoster virus	not specified	lead to the inactivation of the NF-κB pathway by inhibiting SCF^β-TrCP^-mediated IκBα degradation via interaction with β-TrCP	-	[204]

### 6.4. Modulation of β-TrCP Activity by Subcellular Localization

The regulation of the subcellular localization of β-TrCPs is another mechanism to control the β-TrCP-dependent degradation of their target proteins. For example, in glioblastoma cell lines and patient-derived tumor neurospheres, the mislocalization of β-TrCP1 in the nucleus has been reported to uncouple PH domain leucine-rich repeat-containing protein phosphatase 1 (PHLPP1)/the AKT negative feedback loop [209]. Consistent with this, the restoration of β-TrCP1 in the cytoplasm rescues the regulation of PHLPP1 stability by AKT. The nuclear localization of MEK also contributes to the enhanced nuclear localization of β-TrCP [210]. The sequestration of β-TrCP in the nucleus results in the stabilization of YAP in KRAS mutant cancer cells. In addition, the inhibition of mutant KRAS triggers MEK nuclear transportation, leading to KRAS-targeted drug resistance [210].

## 7. Targeting β-TrCP in Cancer

### 7.1. β-TrCP as a Target for Cancer Treatment

Since β-TrCPs upregulates the NF-κB activities that are important for cancer cells’ survival, targeting β-TrCPs has been suggested as a potential effective means to treat cancer [17]. Early evidence that β-TrCPs is a potential target to treat cancer has been demonstrated in several human breast cancer cells. The inhibition of β-TrCPs by either siRNA (Table 5) or a dominant negative β-TrCP^ΔF^ mutant inhibits the growth and survival of human breast cancer cells and augments the cytotoxic effects of anticancer drugs including doxorubicin, tamoxifen, and paclitaxel [211]. In addition, the stable expression of β-TrCP1^ΔF^ in murine myeloma cells has been reported to reduce myeloma cell growth and survival in mice independent of the host immune status [212]. The administration of an IκB-ubiquitin ligase inhibitor, pyrrolidine dithiocarbamate (PDTC) [213], to wildtype β-TrCP1 myeloma tumor-bearing mice reduces tumor burden in the bone. The transgenic expression of β-TrCP2^ΔF^ in mouse skin results in a decrease in UVB-induced edema, hyperplasia, and inflammatory response and an increase in UVB-induced apoptosis [214]. These results suggest that targeting β-TrCP activity provides a therapeutic opportunity, at least in specific types of cancer.

As mentioned earlier, the phosphorylation of β-TrCP may contribute to its stability in TNBC cells [27]. A small-molecule kinase inhibitor, PI-103, targeting PI3K/mTOR, reduces the levels of β-TrCP in a series of TNBC cells and inhibits cell viability. The β-TrCP target proteins including cyclin E and MYC are downregulated by PI-103 treatment in a dose-dependent manner. In addition, siRNA-based knockdown of β-TrCP1 markedly reduces the proliferation of TNBC cells.

In prostate cancer cells, the knockdown of β-TrCP1/2 results in a reduction of cancer cell growth both in vitro and in vivo [215]. The depletion of β-TrCP1/2 induces aryl hydrocarbon receptor (AhR) expression in a ligand-independent manner. How does β-TrCP1/2 depletion induce AhR and its consequences remain to be determined.

Consistent with a recent report [216], the silencing of β-TrCP1/2 induces apoptosis by upregulating BCL2-interacting mediator of cell death extra-long (BimEL) in both gefitinib-sensitive and gefitinib-resistant NSCLC cells [217]. The silencing of RSK1/2, the kinase that phosphorylates the phospho-degron of BimEL, also induces BimEL-mediated apoptosis.

**Table 5:** The effects of β-TrCP silencing in cancer.

β-TrCP Paralogue	Silencing	Cancer Cells	Effect of β-TrCP Inhibition
β-TrCP1	siRNA	TNBC	reduces the proliferation of TNBC cells [27]
β-TrCP1	shRNA	Leukemia	reverses JAK2-inhibitor-mediated β-catenin downregulation [168]
β-TrCP1/2	siRNA	NSCLC	induces apoptosis through upregulation of BimEL [217]
β-TrCP1/2	shRNA	Prostate cancer	inhibits prostate cancer cell growth both in vitro and in vivo by inducing the aryl hydrocarbon receptor (AhR) [215]

### 7.2. Small Molecule Compounds That Modulate β-TrCP Activity

Small-molecule compounds have also been found to inhibit β-TrCPs’ function either directly or indirectly (Table 6). An example of a β-TrCP-specific inhibitor has been developed from a ubiquitin-based engineered inhibitor for β-TrCP2 using the phage display technique [218]. Interestingly, the ubiquitin-based inhibitors competitively bind on the SKP1-F-box interface to block CUL1 binding to the same site, resulting in the inhibition of ligase activity. Further engineering of inhibitors results in developing a highly specific inhibitor for β-TrCP2, while it binds very weakly to β-TrCP1 and does not bind to other E3 ligases tested. Further clinical development of this inhibitor remains to be disclosed. For example, erioflorin interferes with the interaction between β-TrCP1 and programmed cell death protein 4 (PDCD4) and stabilizes PDCD4 protein levels with a concomitant alteration of the cell cycle progression and suppression of the cell proliferation of various cancer cell lines [219].

A recent study reported that a small molecule enhances the β-TrCP–β-catenin interaction [247]. As these kinds of drugs target naturally occurring protein–protein interactions, the molecular glue strategy may provide an alternative method to interfere with hard-to-target proteins [247]. However, the clinical implications of these small molecules remain to be determined.

Targeting cancer cells based on the Warburg effect, metabolic shifting to aerobic glycolysis, by natural-product-based energy restriction-mimetic agents (ERMAs), is a new potential cancer therapy strategy [248,249,250]. Thiazolidinediones (TZDs) have been developed as selective ligands for peroxisome-proliferator-activated receptor gamma (PPARγ) and have been identified as a novel class of ERMAs [251]. TZDs activate the β-TrCP1-mediated proteolysis of its target proteins such as β-catenin, cyclin D1, and SP1 via an increase in the β-TrCP1 expression level [245,246,252]. When glucose is deprived, TZDs activate silent information regulator 1 (SIRT1), AMP-activated protein kinase (AMPK), and ER stress [251]. In addition, TZDs upregulate β-TrCP1 through protein stabilization in an SIRT1-dependnet manner. The energy restriction by TZDs induces apoptosis via β-TrCP1-mediated proteasomal degradation and transcriptional repression in cancer cells [251]. Since SIRT1 is an NAD^+^-dependent deacetylase, the potential involvement of acetylation in the regulation of β-TrCP1 stability remains to be elucidated.

Hydroquinone (HQ) induces the demethylation of the Forkhead box protein P3 (*FOXP3*) gene, resulting in FOXP3 gene expression in U937 cells [155]. As a result, FOXP3 induces the expression of miR-183, leading to a reduction in β-TrCP1 mRNA stability. The downregulation of β-TrCP1 results in the upregulation of its target SP1 expression in U937 cells. SP1-induced ADAM17 and LYB contributes to the proliferation and clonogenicity of U937 cells.

The stability of the cullin family of proteins, including β-TrCP, is regulated by protein neddylation [253]. Recently, MLN4924/pevonedistat, the first-in-class inhibitor of the E1 NEDD8-activating enzyme (NAE), has been reported to block the β-TrCP1-dependent ubiquitination and degradation of mitofusin 1 (MFN1) [237]. MLN4924 inactivates β-TrCP1 via cullin neddylation at S85/86/90.

As mentioned earlier, JAK2 negatively regulates β-TrCP activity in both human leukemia Jurkat cells and human erythroleukemia HEL cells [168]. The knockdown of β-TrCP1 by shRNA results in reversing the downregulation of β-catenin in the presence of the JAK2 inhibitor, AG490. However, the role of β-TrCP1 in leukemia remains elusive.

## 8. Conclusions

β-TrCP1/2 function as either oncogenes or tumor suppressors in a cellular-context-dependent manner. Compared to the long lists of β-TrCP1/2 substrates and their functions, upstream effectors that regulate the expression of mRNAs and the protein stability, function, and localization of β-TrCP1/2 have not been well established yet. Since evidence suggests that β-TrCP1/2 are potential targets to treat certain types of cancers, further studies on the transcriptional and post-translational modulation on β-TrCP1/2 warrant the development of new therapeutic entities to overcome malignant diseases.

## Figures and Tables

**Table 2:** Upstream effectors regulating β-TrCP activity.

Effector	β-TrCP Isoform	Biological Consequences	Role in Cancer	Ref.
**Protein (Also Known as)**				
β-TrCP1	β-TrCP2	decreases the β-TrCP2 level and suppresses cell growth of human cancer cells	suppressive	[116]
β-TrCP2	β-TrCP1	leads to the phosphorylated β-TrCP1 degradation upon glucose deprivation, resulting in autophagy inhibition and inducing human TNBC cell growth	promoting	[116]
ACTL6A (BAF53A)	not specified	decreases level of β-TrCPs and promotes human glioma xenograft tumor growth by inducing YAP stabilization	promoting	[117]
AKT	β-TrCP1 [73]	increases the stability of *BTRC* mRNA by blocking GSK3β in a TCF1-mediated transcription-dependent manner [88]	promoting	[73,88]
AMPK	not specified	upregulates β-TrCP activity to degrade PRLR in response to energy deprivation in mouse mammary epithelial cells	-	[118]
ATM	β-TrCP1	enhances the proteasomal degradation of β-TrCP2 and MDM2, leading to G2/M cell cycle arrest to promote DNA repair in response to DNA damage in human cancer cell lines	-	[115]
BRAF^V600E^	β-TrCP2	enhances β-TrCP expression in melanocytes and increases IKK activity, leading to the activation of the NF-κB transcription factor in human melanoma cells	promoting	[119]
CD147	not specified	inhibits NRF2 degradation by promoting AKT activation, leading to temozolomide resistance in human glioma cells	promoting	[120]
CD166	not specified	induces MCAM degradation through the AKT pathway in a human HCC cell line	suppressive	[121]
CENP-W	β-TrCP1	enhances β-TrCP1 oncogenicity by promoting its nuclear accumulation in HEK293 cells	promoting	[122]
CSN6	not specified	decreases the level of β-TrCPs and increases CRC tumor growth by inducing β-catenin stability in human CRC cells	suppressive	[123]
ERK2	β-TrCP1	induces the destabilization of β-TrCP1 in HEK293T cells	-	[124]
FAF1	not specified	acts as a scaffold protein for β-catenin and β-TrCP to facilitate the β-TrCP-mediated degradation of β-catenin in human breast cancer cells	suppressive	[125]
FBXW8	β-TrCP1	induces β-TrCP1 degradation and promotes cell migration through β-catenin accumulation in an MAPK signaling-dependent manner in human breast cancer cells	promoting	[126,127]
IGF2BP1 (CRD-BP)	β-TrCP1	binds to the coding region of *BTRC* mRNA and consequently increases β-TrCP1 expression in a β-catenin/TCF-signaling-dependent manner in HEK293T cells	promoting	[66]
JNK	β-TrCP1	upregulates *BTRC* mRNA in response to extracellular stresses in HEK293T cells	-	[114]
JNKK2	β-TrCP1	upregulates *BTRC* mRNA in response to extracellular stresses in HEK293T cells	-	[114]
MKK6	β-TrCP1	upregulates *BTRC* mRNA in response to extracellular stresses in HEK293T cells	-	[114]
mTORC2	not specified	induces β-TrCP stabilization in human TNBC cells	promoting	[27]
NOTCH1	not specified	increases YAP1 stability by inhibiting β-TrCP-mediated degradation in human TNBC cells	promoting	[128]
OTUD5	β-TrCP1	deubiquitinates and stabilizes β-TrCP1, leading to activation of the mTOR signaling pathway through β-TrCP1-mediated DEPTOR degradation in human cancer cells	promoting	[129]
P38	β-TrCP1	upregulates *BTRC* mRNA in response to extracellular stresses in HEK293T cells	-	[114]
RASSF1A	β-TrCP1/2	inhibits β-TrCP1/2 activity, leading to an increase in the FBXO5 (EMI1) level, which blocks APC/C activity during the G1-to-S transition in HeLa cells	suppressive	[130]
RASSF1C	β-TrCP1	binds to β-TrCP1 to inhibit β-TrCP1-mediated β-catenin degradation in HeLa cells	promoting	[131]
RBX2 (SAG)	β-TrCP1	ubiquitylates and degrades β-TrCP1 and may play anti-apoptotic roles in human cancer cells	promoting	[132]
PDGF	not specified	induces CSN6 expression through the PDGFR/PI3K/AKT pathway, leading to a decrease of β-TrCP by increasing its ubiquitination and degradation in rat pulmonary arterial smooth muscle cells	-	[133]
PHF19	not specified	interacts with β-TrCP to prevent the ubiquitination and degradation of GLI1, leading to the activation of Hedgehog (Hh) signaling in human HCC cells	promoting	[134]
RPS27L	β-TrCP1	increases the protein half-life of β-TrCP1, leading to activation of mTORC1 and reducing autophagy in human HCC cells	promoting	[134]
SIRT1	β-TrCP1	induces β-TrCP1 degradation in human cancer cells	-	[135]
SKP2	β-TrCP1	interacts with the F-box motif of β-TrCP1 and induces its degradation in human prostate and breast cancer cells	-	[136]
SOX9	β-TrCP1	associates with β-TrCP1 to promote its degradation, leading to the stabilization of GLI1 in human pancreatic cancer cells	promoting	[137]
SRC	not specified	inhibits β-TrCP activity, induced by the Hippo tumor suppressor pathway, to accumulate TAZ in HEK293 cells	promoting	[138]
SMURF1	not specified	increases the protein stability of β-TrCP by reducing the autoubiquitination of β-TrCP in human liver cancer cells	suppressive	[139]
SMURF2 (UBCH5)	β-TrCP1	induces the polyubiquitination and degradation of β-TrCP1 to enhance mutant KRAS stabilization in human cancer cells	promoting	[140]
TSPAN15	β-TrCP1	binds to β-TrCP1 to enhance p-IκBα ubiquitination and degradation, leading to the expression of NF-κB target genes including ICAM1, VCAM1, uPA, MMP9, TNFα, and CCL2 in human esophageal squamous cell carcinoma cells	promoting	[141]
USP24	not specified	directly deubiquitinates and stabilizes β-TrCP in a human macrophage cell line, leading to lung cancer metastasis	promoting	[142]
USP47	β-TrCP1/2	binds to β-TrCP1/2 and induces human cancer cell growth by an unknown mechanism	promoting	[143]
WBP2	β-TrCP1	increases the stability of *BTRC* mRNA, leading to IκBα degradation and subsequent activation of NF-κB, leading to the migration and invasion of human TNBC cells	promoting	[144]
WNT/β-catenin	β-TrCP2	downregulates *FBXW11* transcription in HEK293T cells	-	[113]
WNT1/2	β-TrCP1	increases the stability of *BTRC* mRNA by blocking GSK3β in a TCF1-mediated transcription-dependent manner in COS7 monkey kidney cells	promoting	[68]
**Nucleic acids**				
*circHIPK3*	β-TrCP1	enhances β-TrCP1-mediated ELAVL1 degradation to increase the p21 level, leading to cardiac senescence in mice	-	[145]
*circPVT1*	not specified	binds to the coding region of β-TrCP and blocks the interaction between β-TrCP and MYC, leading to the progression of nasopharyngeal carcinoma through inducing MYC stabilization in human nasopharyngeal carcinoma cells	promoting	[146]
*LINC00460*	β-TrCP1	inhibits β-TrCP11-ELAVL binding, resulting in the inhibition of ELAVL ubiquitination and degradation, which may contribute to human cutaneous squamous cell carcinoma progression	promoting	[147]
*LINC00941*	β-TrCP1	enhances SMAD4 stability by competitive binding with β-TrCP1, leading to EMT in human CRC cells	promoting	[148]
*LINC00942*	β-TrCP1	binds to MSI2 to prevent β-TrCP1-mediated degradation, leading to the stabilization of *MYC* mRNA in human gastric cancer cells	promoting	[149]
*miR-10a*	β-TrCP1	binds to the 3′-UTR of β-TrCP1 and degrades its mRNA	-	[150]
*miR-106b-25*	β-TrCP2	binds to the 3′-UTR of *FBXW11* mRNA and degrades it to induce SNAIL expression and enhance human NSCLC cell migration and invasion	promoting	[151]
*miR-135b*	β-TrCP1	binds to the 3′-UTR of *BTRC* mRNA and degrades it to promote lung cancer metastasis [152]	promoting	[152]
*miR-182*	β-TrCP2	binds to the 3′-UTR of β-TrCP2, degrades its mRNA, and promotes the proliferation and migration of human pancreatic cancer cells [153] and head and neck cancer cells [154]	promoting	[153,154]
*miR-183*	β-TrCP1	reduces *BTRC* mRNA stability in response to hydroquinone [155] and quinacrine [156] in U937 cells	promoting [155] suppressive [156]	[155,156]
*miR-193a-3p*	β-TrCP1	binds to the 3′-UTR of *BTRC* mRNA and degrades it in human glioma cell lines	promoting	[157]
*miR-221*	β-TrCP2	promotes cell growth and cell cycle progression and inhibits apoptosis in human osteosarcoma cells	promoting	[158]
*miR-224*	β-TrCP1	promotes the migration and invasion of human CRC cells	promoting	[159]
*miR-324-5p*	β-TrCP1	reduces the expression of the *BTRC* mRNA level to suppress the migration and invasion of human multiple myeloma cells	suppressive	[160]
*SLC7A11-AS1* (lncRNA)	β-TrCP1	interacts with the F-box motif of β-TrCP1 to prevent the ubiquitination and degradation of NRF2, promoting gemcitabine-resistance in human pancreatic cancer cells	promoting	[161]
**Endogenous small molecules**				
Androgen (dihydrotestosterone (DHT))	not specified	decreases the level of β-TrCPs with a concomitant increase in the REST protein in human prostate cancer cells	promoting	[162]

**Table 3:** Proteins that regulate β-TrCP by protein–protein interactions.

Protein	β-TrCP Isoform	MoA	Biological Consequences	Roles in Cancer
14-3-3ζ (YWHAZ)	not specified	competitive binding to β-catenin	increases β-catenin stability in lung cancer cells [179]	promoting
AP2-β	not specified	binding to β-catenin and β-TrCP	increases β-catenin degradation to inhibit cervical cancer cell proliferation [180]	suppressive
ERα	not specified	binding to β-catenin	triggers β-catenin-β-TrCP binding to promote β-catenin degradation [181]	suppressive
RASS5 (NORE1A)	β-TrCP1	direct binding to β-TrCP1	activates β-TrCP1-mediated β-catenin degradation, but not IκB in an RAS-dependent manner [182]	suppressive
TRIB2	not specified	direct binding to β-TrCP	enhances co-localization of β-TrCP and β-catenin to degrade β-catenin [183]	suppressive
TRIB3	not specified	binding to TAZ	competes with β-TrCP binding to stabilize TAZ, leading to radiotherapy resistance in ESCC cells [184]	promoting
TRIM9	β-TrCP1/2	direct binding to β-TrCP	inhibits NF-κB activation [185]	-
TRIM67	not specified	direct binding to β-TrCP	inhibits TNFα-triggered NF-κB activation [186]	-
UBTD1	not specified	direct binding to β-TrCP	enhances β-TrCP-dependent YAP degradation [187]	-

**Table 6:** Small-molecule compounds affecting the function of β-TrCP.

Small Molecule	Known Targets	Functions	Ref.
ABT-199/WEHI-539 combination	BCL2/BCL2L1 (respectively)	reduces the expression of β-TrCP mRNA, by an unknown mechanism, which increases SP1 expression, leading to the induction of NOXA and SP1-/NOXA-axis-mediated MCL1 degradation in leukemia cells	[220]
Acyclovir	β-TrCP1	binds to the WD40 domain of β-TrCP1 and inhibits its ligase activity	[221]
Aspirin	COX1/2	induces the expression of β-TrCP to attenuate YAP and β-catenin expression, leading to the reduction of the docetaxel and vinorelbine resistance of TNBC cells	[222]
AZD8055	mTOR	induces β-TrCP degradation, leading to an increase of PD-L1 stability in NSCLC cells	[67]
Bergenin	unknown	reduces β-TrCP to induce NRF2 stabilization, leading to the inhibition of the oxidative stress and ECM generation in glomerular mesangial cells	[223]
CHIR-99021	GSK3α/β	induces an increase in the β-TrCP1 levels with a concomitant increase of the MYC level in TNBC cells	[27]
CIB-6	STAT3	induces the expression of β-TrCP by inhibiting IFN-α-induced STAT3 activation, leading to a reduction of HCC tumor growth and cell motility	[224]
Corosolic acids	unknown	induces the expression of β-TrCP by an unknown mechanism, leading to the induction of cell apoptosis by inducing YAP degradation	[225]
Curcumin	P300/HDAC	promotes the interaction between NRF2 and GSK3α/β-TrCP, leading to anticancer effects	[226]
Decitabine	DNA methyltransferase	induces the expression of the β-TrCP mRNA by inhibiting the methylation of the β-TrCP promoter, leading to IκBα degradation and NF-kB activation in IFN-γ^+^CD4^+^ T cells	[227]
Diindolylmethane, 3,3′-	AR	enhances β-TrCP expression, leading to NF-κB activation in gastric cancer-derived MSCs	[228]
Dihydrocapsaicin	TRPV1	upregulates β-TrCP1 to inhibit the β-catenin pathway	[229]
Doxorubicin	DNA topo II	upregulates β-TrCPs by an unknown mechanism, leading to the degradation of SP1 and the subsequent downregulation of *ADAM10* mRNA expression in MCF7 cells	[230]
Echinomycin	HIF1	inhibits the expression of β-TrCPs, leading to reduced lung adenocarcinoma and lymphoma tumor growth by blocking the expression of the MYC and HIF-1α proteins	[231]
Erioflorin	β-TrCP1	inhibits the interaction between β-TrCP1 and PDCD4 alters cell cycle progression and suppresses the cell proliferation of various cancer cell lines	[219]
Euphorbiasteroid	AMPK	increases β-TrCP with a concomitant reduction of p-GSK3β S9 and exerts anticancer activity in NSCLC cells	[232]
Fisetin	SIRTs	upregulates β-TrCPs by an unknown mechanism, leading to the degradation of β-catenin in human melanoma cells	[233]
Gallic acid	-	induces *BTRC* mRNA expression, leading to the degradation of BCR-ABL in human leukemia cells	[234]
GS143	β-TrCP1–p-IκBα interaction	inhibits the interaction between β-TrCP1 and p-IκBα to suppress IκBα ubiquitylation	[235]
Hydroquinone	Melanin synthesis	induces the malignant progression of U937 cells through the FOXP3/miR-183/β-TrCP1/SP1/LYN axis	[155]
INK128	mTOR	induces β-TrCP degradation, leading to an increase of PD-L1 stability in NSCLC cells	[67]
LY294002	PI3Kα/δ/β	induces the expression of β-TrCPs, by an unknown mechanism, leading to a reduction in prostate cancer growth by reducing the REST protein’s stability	[236]
MLN4924 (pevonedistat)	NEDD8	inhibits β-TrCPs’ ubiquitinylation and degradation via blocking neddylation, leading to mitochondrial fusion by inducing MFN1 accumulation, resulting in anticancer effects	[237]
		suppresses the growth of liver cancer cell through IκBα degradation via the accumulation of β-TrCPs	[238]
Metformin	AMPK	upregulates β-TrCPs in HNSCC cells by an unknown mechanism	[239]
MK2206	AKT	increases the β-TrCP1 level with a concomitant increase in the MYC level in TNBC cells	[27]
OSU-CG5	Energy metabolism	increases β-TrCP, leading to the degradation of cyclin D1 and SP1 in CRC cells	[240]
PF4708671	P70S6K	induces β-TrCP degradation, leading to an increase in PD-L1’s stability in NSCLC cells	[67]
PHAR	β-TrCP1/NRF2 interaction	inhibits the interaction between β-TrCP and NRF2, leading to anti-inflammatory responses in mouse liver	[241]
PI3Kα inhibitors	PI3Kα	reduces the β-TrCP1 level with a concomitant reduction of the MYC level in TNBC cells	[27]
PI3K/mTOR dual-inhibitors	PI3K/mTOR	reduces the β-TrCP1 level with a concomitant reduction of the MYC level in TNBC cells	[27]
Piceatannol	SYK	induces the mRNA stability of β-TrCP by reducing the miR-183 transcript level by inactivating the AKT-mediated expression of the FOXP3 transcription factor	[242]
Quinacrine	PLA2	induces apoptosis in U937 cells through FOXP3/miR-183/β-TrCP1/SP1 axis-mediated BAX upregulation	[156]
		enhances the binding between p-CHEK1/2 and β-TrCP and promotes their degradation, leading to cell death in P53-negative cancer cells	[243]
Rapamycin	mTORC1	increases the β-TrCP1 level with a concomitant increase of the MYC level in TNBC cells	[27]
Retinoic acid, all-*trans*	RAR/RXR	induces REST degradation in neuronal cells by increasing the expression of β-TrCP mRNA	[244]
STG28	PPARγ	Reduces the levels of β-catenin and cyclin D1 by inducing β-TrCP independently of PPARγ	[245,246]

## Supplementary Materials

The following Supporting Information can be downloaded at: https://www.mdpi.com/article/10.3390/cancers15174248/s1, Table S1: Substrates of β-TrCP and their biological functions.
