# Supplementary material for: Beta-Transducin Repeats-Containing Proteins as an Anticancer Target
Source: Cancers (Basel). 2023 Aug 24;15(17):4248. doi: 10.3390/cancers15174248 (PMC10487276; doi:10.3390/cancers15174248)
Supplement: Supplementary file 1 [file cancers-15-04248-s001.zip › cancers-2560323-supplementary.pdf]

Supplementary Table S1

for

Beta-Transducin Repeats-Containing Proteins as an Anticancer Target

Dong Joon Kim, Yong Weon Yi and Yeon-Sun Seong

Table S1. Substrates of β-TrCP and their biological functions.

Substrate (aka)	Prior phosphorylation by	Biological functions induced by β-TrCP-mediated degradation	β-TrCP paralogue	Role in cancer
β-catenin	CDK2 [1]	• fine tunes the β-catenin level in G1 phase [1]	β-TrCP1 [1]	suppressive
	CK1α [2]	• induces APC-independent β-catenin degradation [2]	not specified [2]	
	GSK3β [3–8]	• inhibits TCF1α-dependent transcription [5]<br>• suppresses metastatic potency [8]	β-TrCP1 [3,5–7]<br>β-TrCP2 [4]	
	-	• inhibits HCC tumor growth [9]	β-TrCP1/2 [9]	
	IKKβ [10]	• regulates adipocyte and osteoblast differentiation [10]	not specified [10]	-
	-	• induces APC-independent β-catenin degradation in autophagy-dependent manner [11]	not specified [11]	
	NEK2 [12]	• inhibits monopolar spindle in mitosis [12]	not specified [12]	
β-TrCP1 (FBXW1A)	AMPK [13]	• inhibits autophagy and promotes cell growth by activating mTORC1 [13]	β-TrCP2 [13]	promoting
β-TrCP2 (FBXW11)	-	• suppresses cell growth [13]<br>• induces MDM2 degradation, G2/M cell cycle arrest, and DNA repair upon DNA damage [14]	β-TrCP1 [13]	suppressive
AEBP2	-	• inhibits cell proliferation and cisplatin resistance in ovarian cancer cells [15]	β-TrCP2 [15,16]	suppressive
AMER1	-	• not determined	β-TrCP2 [16]	-
ARID1A	IKKβ [17]<br>ATM [18]	• induces prostate cancer progression [17]<br>• associated with poor patient prognosis [18]	not specified [17,18]	promoting
ATF4	CK1δ/ε [19]	• upregulates cAMP-responsive element (CRE)-dependent transcription of somatostatin promoter [20]<br>• undergoes cell cycle progression from ATK4-mediated G0/G1 arrest [19]	β-TrCP1 [20]<br>β-TrCP2 [16,19]	-
AUF1	GSK3β/PKA [21]	• increases the stability of mRNAs containing AU-rich elements upon IL-1-induced MAPK activation [21]	β-TrCP1 [21]	-
BCL10	IKKβ [22]	• terminates NF-κB signaling in T cells after antigen receptor stimulation [22]	β-TrCP1 [22]	promoting

Substrate (aka)	Prior phosphorylation by	Biological functions induced by β-TrCP-mediated degradation	β-TrCP paralogue	Role in cancer
BimEL	RSK1/2 [23]	• inhibits apoptosis [23]	β-TrCP1/2 [23]	promoting
	AURKA [24]	• promotes cell survival during mitosis in cancer cells [24]	β-TrCP1 [24]	promoting
BMI1	-	• inhibits proliferation of HDFs [25]	β-TrCP1 [25]	suppressive?
BORA	PLK1 [26–28]	• induces the mitotic progression of cell cycle [26–28]	β-TrCP1 [26,27]	-
BCR-ABL	-	• decreases MMP2 and MMP9 mRNA expression in BCR-ABL+ K562 cells upon gallic acid treatment [29]	β-TrCP1 [29]	-
BTG1 & BTG2	-	• promotes growth of cancer cells [30]	β-TrCP1 [30]	promoting
Caspase-3, pro-	-	• protects cells from apoptosis [31]	β-TrCP1/2 [31]	promoting
CCND1 (cyclin D1 or BCL1)	-	• inhibits growth of hepatocellular carcinoma cells [32]	β-TrCP1 [32]	suppressive
CCNF (cyclin F)	CKIIα [33]	• induces timely mitotic progression via inducing B-MYB transcriptional activity [33]	β-TrCP1/2 [33]	-
CD4	-	• avoids superinfection interference [34]	β-TrCP1/2 [35]	-
CDC20 (CDH1)	CDK2-Cyclin A/PLK1 [36]	• induces G1 to S cell-cycle transition [36]	β-TrCP1 [36]	-
CDC25A	PLK3/GSK3β [37]<br>CHEK1/GSK3β/CK1α [37,38]	• delays cell-cycle progression to prevent genomic instability in response to DNA damage [39]	β-TrCP1/2 [16,39,40]	suppressive
	NEK11 [41]	• induces the DNA damage-induced G2/M arrest [41]	not specified [41]	
	CK1ε [42]	• induces cell cycle arrest [42]	not specified [42]	-
CDC25B	JNK1/2 [43,44]	• DAG and PEST-like sequence-dependent Cdc25B ubiquitylation [44]	β-TrCP1 [45]<br>β-TrCP1/2 [44]	-
	-	• regulates metaphase to anaphase transition [46]	not specified [46]	
CDKN2A (p19ARF)	S6K [47]	• promotes cell proliferation under nutrient-rich conditions [47]	β-TrCP2 [47]	-
CELF6	-	• induces tumor growth through reducing CELF6-mediated the stability of P21 transcript [48]	not specified [48]	promoting
CEP68	PLK1 [49]<br>NEK2 [50]	• induces centriole disengagement, separation, and duplication [49]	β-TrCP1 [49]<br>β-TrCP1/2 [50]	-
CHD1	GSK3β [51]	• inhibits cancer progression through suppressing gene transcription of NF-kB signaling pathway in PTEN-intact cancers [51]	not specified [51]	suppressive
CHEK1 (CHK1)	RSK1/2 [52]<br>AMPK [53]	• induces cancer cell survival upon glucose deprivation in breast cancer cells [52]	not specified [52,53]	promoting

Substrate (aka)	Prior phosphorylation by	Biological functions induced by β-TrCP-mediated degradation	β-TrCP paralogue	Role in cancer
		• induces cancer cell survival in response to glucose deprivation [53]		
CHEK1 & CHEK2 (CHK2)	-	• induces cell death in P53-negative cancer cells in response to quinacrine [54]	not specified [54]	suppressive
CLSPN	-	• releases DNA-damage-induced cell cycle arrest leading to the onset of mitosis [55]<br>• restore CDK1 activity upon recovery from DNA damage [55]	β-TrCP1/2 [55]	-
	GSK3β [56]	• inhibits CHEK1 activation upon DNA damage insults [56]	not specified [56]cls	promoting
Cortactin (EMS1)	ERK [57]	• reduces the lung epithelial barrier function [57]	β-TrCP1 [57]	-
CREB3L3 (CREB-H)	-	• regulates lipid homeostasis and gluconeogenesis in liver cells [58]	β-TrCP1 [58]	-
CTNND2 (δ-catenin 2)	GSK3β [59]	• inhibits angiogenesis through reducing HIF-1 stabilization, and reduces -catenin-mediated oncogenic signals in prostate cancer cells [59]	β-TrCP1 [59]	suppressive
CYLD	IKKα/IKKβ [60]	• induces osteoclast differentiation through NF- B activation [60]	β-TrCP1/2 [60]	-
DDIT4 (REDD1)	GSK3β [61]	• activates the mTORC1 signaling [61]	β-TrCP1 [61]	promoting
DEC1	CK1α [62]	• induces G2 checkpoint recovery upon DNA damage [62]	β-TrCP1/2 [62]	suppressive
DEPTOR	mTOR/CK1α [63–65]<br>S6K1/RSK1 [66]	• activates the mTOR signaling [63–66]	β-TrCP1/2 [64,66]	promoting
DLG1 (SAP97)	-	• not determined	β-TrCP1 [67]	-
DLG5	-	• promotes HCC tumor growth [53]	not specified [53]	promoting
DMRT1 (DMT1)	-	• regulates the mitosis-meiosis transition in mouse male germ cells [68]	β-TrCP1/2 [68]	-
DNMT1	-	• inhibits cell migration and tumor growth in HCC cells [69]	not specified [69]	suppressive
ELAVL1 (HuR)	PKCα/IKKα [70]	• reduces the expression of oncogenic proteins in response to glucose depletion or glycolysis inhibitors [70]	β-TrCP1 [70]	suppressive
ELF3 (ESE-1)	-	• not determined	β-TrCP1 [71]	-
ERBIN (ERBB2IP)	-	• inhibits KRASG12D-induced papillomagenesis through inducing senescence and autophagy [72]	β-TrCP [72]	suppressive
EZH2	JAK2 [73]	• inhibits tumor growth through reducing H3K27 trimethylation activity [73]	β-TrCP1 [73]	suppressive
FANCM	PLK1 [74]	• not determined	β-TrCP1/2 [74]	-
FBXO5 (EMI1)	Cyclin/CDC2 [75,76]	• activates the anaphase-promoting complex (APC) in mitosis [76]	β-TrCP1/2 [75,76]	-

Substrate (aka)	Prior phosphorylation by	Biological functions induced by β-TrCP-mediated degradation	β-TrCP paralogue	Role in cancer
	PLK1 [77]			
FBXW2 (FBW2)	-	• promotes lung cancer growth through inhibiting FBXW2-mediated SKP2 degradation, leading to degradation of SKP2 targets including P21, P27, P130, and FOXO1 [78]	β-TrCP1 [78]	promoting
Fibronectin (FN)	-	• promotes FN matrix turnover by lysosomal degradation [79]	β-TrCP1/2 [79]	-
FGD1	GSK3β [80]	• inhibits the filopodia formation by negatively regulates the CDC42 GEF activity of FGD1 [80]	β-TrCP1 [80]	-
FGD3	GSK3β [81]	• stimulates cell migration [81]	β-TrCP1 [81]	
FNIP2	CK1 [82]	• induces renal tumor growth through lysosomal dissociation of FLCN and subsequent lysosomal association of mTOR, leading to proliferation of renal cancer cells [82]	β-TrCP1 [82]	promoting
FOXN2 (HTLF)	RSK2 [83]	• promotes tumorigenesis and radioresistance in lung cancer [83]	β-TrCP1 [83]	promoting
FOXO3 (FOXO3A)	IKKβ [84]	• promotes tumorigenesis and tumor growth [84]	β-TrCP1 [84]	promoting
	IKKβ [85]	• induces paclitaxel resistance [85]	not specified [85]	promoting
FOXP3	GSK3β [86]	• enhances the suppressive function of Treg cells in response to AREG [86]	not specified [86]	promoting
GBF1	CK2 [87]	• induces Golgi inheritance and accurate cell division [87]	β-TrCP [87]	-
GHR	-	• results in GH insensitivity [88]	β-TrCP2 [88]	-
GLI1	AMPK [89]	• inhibits cell growth and colony formation in medulloblastoma a [89]	β-TrCP1 [89]	suppressive
GLI2	GSK3β [90]	• downregulates transcription of target genes that are activated by the Hedgehog signaling pathway [90]	β-TrCP2 [90]	suppressive
GSK3β (GSK3B)	–	• increases β-catenin stability through GSK3β monoubiquitination [91]	not specified [91]	-
HER2 (ERBB2)	Autophosphorylation [92]	• dephosphorylation of HER2 by protein tyrosine phosphatase N18 (PTPN18) promotes HER2 degradation by β-TrCP [92]<br>• inhibits proliferation and invasion [92]<br>• induces HER2+ breast cancer cell apoptosis through inhibiting HER2/PI3K/AKT/mTOR signaling [93]	not specified [92]<br>β-TrCP1/2 [93]	suppressive
HIC1	-	• promotes CRC tumor growth and liver metastasis through inducing SIRT1 expression [94]	β-TrCP2 [PMC8511012]	promoting
HIF-1α	GSK3β [95]	• inhibits proliferation and migration in response to tunicamycin-induced ER-stress [95]	β-TrCP1 [95]	-
HRAS	GSK3β [96]	• inhibits intestinal tumorigenesis [96]	not specified [96]	suppressive

Substrate (aka)	Prior phosphorylation by	Biological functions induced by β-TrCP-mediated degradation	β-TrCP paralogue	Role in cancer
HSF1	PLK1 [97]	• promotes mitotic progression [97]	β-TrCP1 [97]	promoting
IFNAR1	PERK/CK1α [98]	• downregulates p-STAT1 (Y701) and inhibits STAT1-mediated transcription [99]<br>• PERK & CK1α [98]	β-TrCP2 [99,100]<br>not specified [98]	-
IκBα (NFKBIA, IKBA)	IKKβ [4,7,101–108]	• activates NF-κB p50-p65 and p50-c-REL [104]<br>• Blocking β-TrCP2 sensitizes melanoma cells to apoptosis induced by TNFα with cycloheximide [109]<br>• IKKβ adaptor protein-mediated suppression of anti-apoptotic gene expression [108]	β-TrCP1 [103–107]<br>β-TrCP2 [4,106,109]<br>not specified [108]	promoting
IκBβ (NFKBIB, IKBB)	IKKβ [110,111]	• activates NF-κB p50-p65 and p50-c-REL [110]<br>• Blocking β-TrCP2 sensitizes melanoma cells to apoptosis induced by TNFα with cycloheximide [109]	β-TrCP1 [110]<br>β-TrCP2 [4]	promoting
IκBε (NFKBIE, IKBE)	IKKβ [110]		β-TrCP1 [110]	promoting
IL10RA (IL-10R1)	-	• Inhibits JAK/STAT signaling pathway [112]	not specified [112]	-
IRAK1 (IRAK)	-	• releases TAK1-TRAF6 from plasma membrane to cytosol to activate NF-κB in response to IL1 [113]	β-TrCP1/2 [113]	-
IRS1	mTORC1 [114]	• impair growth and survival response to IGF through reducing PI3K/AKT signaling in TSC2 lacking cells [114]	β-TrCP1/2 [114]	-
KMT5A (SET8)	CK1δ [115]	• increases cell proliferation [115]	β-TrCP1 [115]	Promoting
LPCAT1	GSK3β [116]	• reduces the generation of bioactive surfactant phospholipid, dipalmitoylphosphatidylcholine (DPPtdCho) in response to LPS treatment [116]	not specified [116]	-
LPIN1	mTORC1/CK1 [117]	• regulates hepatocyte lipogenesis through inducing SREBP-dependent gene expression and triglyceride synthesis [117]	β-TrCP1/2 [117]	-
LZTS2	CK1δ [118]	• promotes tumorigenesis and metastasis through activation of PI3K/AKT signaling in HCC [118]	not specified [118]	promoting
MCAM	-	• reduces transformative phenotype in HCC cells [119]	not specified [119]	suppressive
MCL1 (BCL2L3)	GSK3β [120,121]	• induces apoptosis and chemosensitization in breast cancer cells [120]	β-TrCP1/2 [120,122]	suppressive
MCT4	-	• inhibits breast tumor growth [123]	not specified [123]	suppressive
MDM2	CK1δ/ε [124]	• inhibits tumorigenesis through inducing p53 protein stability [124,125]	not specified [125]<br>β-TrCP1 [124,126]	suppressive

Substrate (aka)	Prior phosphorylation by	Biological functions induced by β-TrCP-mediated degradation	β-TrCP paralogue	Role in cancer
		• induces senescence in the RAS/NORE1A-dependent manner in NSCLC cells [126]		
	-	• induces G2/M cell cycle arrest to promote DNA repair in response to genotoxic DNA damage [14]	β-TrCP2 [14]	-
MFN1	-	• block mitochondrial fission-to-fission conversion [127]	β-TrCP1 [127]	promoting
MIS18β	-	• inactivates MIS18 complex during metaphase [128]	not specified [128]	-
MST2	-	• not determined	not specified [129]	-
MTSS1	CK1δ [130]	• induces proliferation and invasion in prostate and breast cancer cells [130]	β-TrCP1/2 [130]	promoting
MTSS1L	-	• not determined	β-TrCP2 [16]	-
MXI1	S6K1 [131]	• induces MYC activation and radio-resistance in lung cancer [131]	β-TrCP1/2 [131]	promoting
	-	• induces radioresistance in NSCLC cells [132]	not specified [132]	
NEDD4	CK1δ [133]	• inhibits tumorigenesis by modulating the PTEN/AKT signaling pathway [133]	β-TrCP1 [133]	suppressive
NEF2L3 (NRF3)	-	• inhibits colon cancer proliferation through reducing UHMK1 expression in nuclear [134]	β-TrCP1/2 [PMC5624902]	suppressive
NFKB1 (NF-κB p105)	IKKα/β [135,136]	• liberates NF-κB p50, c-REL, and RELA to translocate into the nucleus for transcriptional activating their target gene [136]	β-TrCP1/2 [136]	promoting
NFKB2 (NF-κB p100)	NIK/IKKα [137]	• induces B cell survival and osteoclatogenesis [137]	β-TrCP1/2 [16,137]	-
NHEJ1 (XLF)	CK1 [138]	• impairs DNA damage-induced NHEJ and V(D)J recombination and leads to genomic instability and tumorigenesis [138]	β-TrCP [138]	suppressive
NRF2 (NFE2L2)	GSK3β [139,140]	• PTEN-mediated GSK3β activation result in inhibiting NRF2 transcriptional activity in KEAP1-independent manner [139,140]	β-TrCP1/2 [16,139,141]	suppressive
	GSK3β [142]	• promotes oxidative stress and poor glucose tolerance through inducing nuclear NRF2 degradation by β-TrCP in the hyperglycemic renal cells in a PHLPP1-dependent manner [142]	not specified [142]	-
	GSK3β [143]	• induce Ca^{2+} influx via activating TRPV1 in response to UVB in human dermal fibroblast [143]	not specified [143]	-
PDCD4	S6K1 [144,145]<br>AKT [146]<br>RSK1 [147]	• results in efficient protein synthesis and cell growth [144]<br>• induces IFNα-mediated eIF4G expression [147]	β-TrCP1 [144,146,147]	promoting

Substrate (aka)	Prior phosphorylation by	Biological functions induced by β-TrCP-mediated degradation	β-TrCP paralogue	Role in cancer
PD-L1	GSK3β [148]	• enhances efficacy of cancer immunotherapy [148]	not specified [148]	suppressive
PER2	CK1ε [149–151]<br>CK1δ [150]	• progressing circadian rhythm [149,152]	β-TrCP1/2 [153,154]	-
PHF10-S	-	• leads to degradation of other components of the PBAF-signatures (BAviaF200, BAF180, and BRD7) [155]	β-TrCP [155]	-
PHLPP1	CK1/GSK3β [156]	• upregulates AKT activity [156,157]	β-TrCP1 [156,157]	promoting
PKD1	-	• activates LPS-induced NF-κB signaling [158]	β-TrCP [158]	-
PKD2 (PC2)	-	• not determined	β-TrCP1/2 [159]	-
PKR	-	• Induces Rift Valley fever virus (RVFV) replication [160]	β-TrCP1/2 [160]	-
PLK1	CDK1 & GSK3β [161]	• delays G1/S phase cell cycle transition [161]	not specified [161]	suppressive
PLK4	Trans-autophosphorylation [162]	• regulates centrioles overduplication [162]	not specified [162]	suppressive
PPP1R15B/CREP	-	• regulates stress-induced inhibitory phosphorylation and cap-dependent translation upon DNA damage through inducing phosphorylation of eIF2α at serine 51 [163]	not specified [163]<br>β-TrCP1/2 [164]	-
PRLR	GSK3β [165]	• negatively regulates the prolactin (PRL)-induced STAT5 signaling [166]	β-TrCP2 [166]	suppressive
PTENα/β	-	• inhibits tumorigenesis through reducing PTENα/β protein stability [167]	β-TrCP2 [167]	suppressive
RAP1GAP	PLK1 [168]	• increases cell proliferation [168]	β-TrCP1/2 [168]	promoting
RAPGEF2	-	• reduces multinucleation [16]	β-TrCP2 [16]	-
RASSF1C	GSK3β [169]	• may involved in DNA damage response upon UV irradiation [169]<br>• β-TrCPs is responsible to RASSF1C degradation upon UV irradiation but not under normal condition [169]	β-TrCP1/2 [169]	-
REST (NRSF)	-	• derepresses the transcription of MAD2L1/MAD2 to activate the spindle checkpoint [170]	β-TrCP1 [170]	promoting
	PIN1 & ERK1/2 [171]	• inhibits terminal neuronal differentiation [171]	β-TrCP [171]	-
	CK1 [172]	• inhibits neuronal death induced by ischemia in rats [172]	β-TrCP1/2 [172]	-
RIPK4	Autophosphorylation [173]	• inhibits the formation of actin stress fibers in keratinocytes [173]	β-TrCP1/2 [173]	-

Substrate (aka)	Prior phosphorylation by	Biological functions induced by β-TrCP-mediated degradation	β-TrCP paralogue	Role in cancer
SET8	CK1δ [115]	• increases cell proliferation [115]	β-TrCP1 [115]	promoting
SMAD3 (MADH3)	-	• terminates the TGFβ-induced SMAD3 transcriptional activity [174]	β-TrCP1 [174]	-
SMAD4 (MADH4, DPC4)	ERK1/2 & GSK3β [175]	• inhibits TGFβ dependent transcription [176]	β-TrCP1 [176]	promoting
SMAD9 (SMAD8, MADH6, MADH9)	-	• not determined	β-TrCP1 [174]	-
SNAIL (SNAI1)	CK1ε [177] /GSK3β [8,178,179]	• upregulates E-cadherin, leading to inhibition of EMT [177]<br>• suppresses metastasis potency [8]	β-TrCP1 [178,179]<br>not specified [8]	suppressive
SP1	GSK3β [180]	• suppresses the transcription of SP1 target genes, such as AR, in response to glucose deprivation [180]<br>• promotes malignant progression of U937 cells by inducing ADAM17 and LYN in response to hydroquinone [181]<br>• induces BAX expression in response to quinacrine through ROS-mediated P38 activation and ERK inactivation, leading to up-regulation of FOXP3-mediated miR-183 expression in U937 cells [182]	β-TrCP1 [180,181]	suppressive
STARD9	PLK1 [183]	• promotes mitotic spindle assembly [183]	not specified [183]	-
STIL	-	• controls the centriole number [184]	β-TrCP1/2 [184]	-
SUN2	CK2 [185]	• maintains nuclear envelope architecture [185]	β-TrCP2 [185]	-
TAZ (WWTR1)	LATS/CK1ε [186]	• inhibits cell proliferation and EMT [186]	not specified [186,187]	suppressive
	LATS/GSK3 [187]	• inhibits cell growth and migration [187]		
	-	• not determined	β-TrCP1/2 [159]	-
TF2L1 (Tfcp2l1)	MK2 [188]	• reduces mouse embryonic stem cell self-renewal [188]	β-TrCP1/2 [188]	-
TIAM1	CK1δ/CK1ε [189]	• leads to DNA damage-induced cell apoptosis through RAC1/JNK activation [189]	not specified [189]<br>β-TrCP1/2 [190]	promoting
TIPE2	TAK1 [191]	• induces activation of TLR4-induced NF-κB signaling [191]	β-TrCP1 [191]	-
TNRC6B	-	• inhibits P-body formation [164]	β-TrCP1/2 [164]	-
TOP2B	CK1δ/ATM [192]	• promotes cancer cell survival in response to topoisomerase II targeting drug teniposide [192]	β-TrCP1/2 [192]	promoting
TP53	IKKβ [193]	• reduces the expression of TP53 target genes [193]	β-TrCP1 [193]	promoting
TTP	P38 [194]	• induces TNF-α mRNA stabilization and radiation pneumonitis [194]	β-TrCP1 [194]	

Substrate (aka)	Prior phosphorylation by	Biological functions induced by β-TrCP-mediated degradation	β-TrCP paralogue	Role in cancer
TRAF3IP2 (ACT1)	-	• desensitizes IL17R signaling in HeLa cells in response to IL17 [195]	β-TrCP1/2 [195]	-
TRF1	-	• increases the formation alternative lengthening of telomeres (ALT)-associated promyelocytic leukemia (PML) bodies in U2OS cells [196]	β-TrCP1 [196]	
TRIB2	-	• not determined	β-TrCP1 [197]	-
TWIST1 (TWIST)	AKT1 [198]	• inhibits EMT in breast cancer cells [198]	β-TrCP [198]	suppressive
	IKKβ [199]	• not determined	β-TrCP1 [199]	suppressive
UHRF1	CK1δ [200]	• not determined	β-TrCP1 [200]	-
ULK1	ERK1/2 [201]	• induces breast cancer metastasis through activation of NLRP3 inflammasome and attenuation of mitophagy [201]	β-TrCP1 [201]	promoting
KDR (FLK1, VEGFR2)	CK1δ [202]<br>GSK3β [203]	• inhibits cell migration and angiogenesis of thyroid cancer cells [202]	β-TrCP1 [202–204]	suppressive
WEE1	PLK1 & CDK1/CDC2 [205,206]<br>PLK1 & CK2 [206]	• roles in G_2/M cell-cycle transition Ref<br>• restore CDK1 activity upon recovery from DNA damage Ref	β-TrCP1/2 [16,205,206]	
XRCC1	-	• confers chemoresistance in lung cancer cells by SIRT1-mediated deacetylation of XRCC1 [207]	β-TrCP2 [207]	promoting
YAP1 (YAP65)	LATS / CK1δ/ε [208]	• reduces transformation activity [208]	β-TrCP [208]	suppressive
YAP1 isoform 2	LATS1/2 [209]	• reduces transformation activity at high cell density [209]	β-TrCP [209]	suppressive
ZNF281	GSK3β [210]	• inhibits colorectal cancer progression [210]	β-TrCP2 [210]	suppressive
ZNRF3	CK1δ [211]	• positively regulates WNT signaling by FZD stabilization [211]	β-TrCP1 [211]	promoting

References for Supplementary Table 1

1. Park, C.S.; Kim, S.I.; Lee, M.S.; Youn, C.; Kim, D.J.; Jho, E.; Song, W.K. Modulation of β-Catenin Phosphorylation/Degradation by Cyclin-Dependent Kinase 2*. *J Biol Chem* **2004**, *279*, 19592–19599, doi:10.1074/jbc.m314208200.

2. Ranes, M.; Zaleska, M.; Sakalas, S.; Knight, R.; Guettler, S. Reconstitution of the Destruction Complex Defines Roles of AXIN Polymers and APC in β-Catenin Capture, Phosphorylation, and Ubiquitylation. *Mol. Cell* **2021**, *81*, 3246-3261.e11, doi:10.1016/j.molcel.2021.07.013.

3. Kitagawa, M.; Hatakeyama, S.; Shirane, M.; Matsumoto, M.; Ishida, N.; Hattori, K.; Nakamichi, I.; Kikuchi, A.; Nakayama, K.; Nakayama, K. An F-box Protein, FWD1, Mediates Ubiquitin-dependent Proteolysis of B-catenin. *Embo J* **1999**, *18*, 2401–2410, doi:10.1093/emboj/18.9.2401.

4. Fuchs, S.Y.; Chen, A.; Xiong, Y.; Pan, Z.-Q.; Ronai, Z. HOS, a Human Homolog of Slimb, Forms an SCF Complex with Skp1 and Cullin1 and Targets the Phosphorylation-Dependent Degradation of IκB and β-Catenin. *Oncogene* **1999**, *18*, 2039–2046, doi:10.1038/sj.onc.1202760.

5. Hart, M.; Concordet, J.-P.; Lassot, I.; Albert, I.; Santos, R. del los; Durand, H.; Perret, C.; Rubinfeld, B.; Margottin, F.; Benarous, R.; et al. The F-Box Protein β-TrCP Associates with Phosphorylated β-Catenin and Regulates Its Activity in the Cell. *Curr Biol* **1999**, *9*, 207–211, doi:10.1016/s0960-9822(99)80091-8.

6. Latres, E.; Chiaur, D.S.; Pagano, M. The Human F Box Protein β-Trcp Associates with the Cul1/Skp1 Complex and Regulates the Stability of β-Catenin. *Oncogene* **1999**, *18*, 849–854, doi:10.1038/sj.onc.1202653.

7. Winston, J.T.; Strack, P.; Beer-Romero, P.; Chu, C.Y.; Elledge, S.J.; Harper, J.W. The SCFβ-TRCP–Ubiquitin Ligase Complex Associates Specifically with Phosphorylated Destruction Motifs in IκBα and β-Catenin and Stimulates IκBα Ubiquitination in Vitro. *Gene Dev* **1999**, *13*, 270–283, doi:10.1101/gad.13.3.270.

8. Tseng, R.-C.; Lee, S.-H.; Hsu, H.-S.; Chen, B.-H.; Tsai, W.-C.; Tzao, C.; Wang, Y.-C. SLIT2 Attenuation during Lung Cancer Progression Deregulates β-Catenin and E-Cadherin and Associates with Poor Prognosis. *Cancer Res* **2010**, *70*, 543–551, doi:10.1158/0008-5472.can-09-2084.

9. Xu, S.; Tong, M.; Huang, J.; Zhang, Y.; Qiao, Y.; Weng, W.; Liu, W.; Wang, J.; Sun, F. TRIB2 Inhibits Wnt/B-Catenin/TCF4 Signaling through Its Associated Ubiquitin E3 Ligases, B-TrCP, COP1 and Smurf1, in Liver Cancer Cells. *Febs Lett* **2014**, *588*, 4334–4341, doi:10.1016/j.febslet.2014.09.042.

10. Sui, Y.; Liu, Z.; Park, S.-H.; Thatcher, S.E.; Zhu, B.; Fernandez, J.P.; Molina, H.; Kern, P.A.; Zhou, C. IKKβ Is a β-Catenin Kinase That Regulates Mesenchymal Stem Cell Differentiation. *Jci Insight* **2018**, *3*, e96660, doi:10.1172/jci.insight.96660.

11. Choi, J.D.; Ryu, M.; Park, M.A.; Jeong, G.; Lee, J.-S. FIP200 Inhibits β-Catenin-Mediated Transcription by Promoting APC-Independent β-Catenin Ubiquitination. *Oncogene* **2013**, *32*, 2421–2432, doi:10.1038/onc.2012.262.

12. Mbom, B.C.; Siemers, K.A.; Ostrowski, M.A.; Nelson, W.J.; Barth, A.I.M. Nek2 Phosphorylates and Stabilizes β-Catenin at Mitotic Centrosomes Downstream of Plk1. *Mol Biol Cell* **2014**, *25*, 977–991, doi:10.1091/mbc.e13-06-0349.

13. Cui, D.; Dai, X.; Shu, J.; Ma, Y.; Wei, D.; Xiong, X.; Zhao, Y. The Cross Talk of Two Family Members of β-TrCP in the Regulation of Cell Autophagy and Growth. *Cell Death Differ* **2020**, *27*, 1119–1133, doi:10.1038/s41418-019-0402-x.

14. Islam, S.; Dutta, P.; Sahay, O.; Santra, M.K. β-TrCP1 Facilitates Cell Cycle Checkpoint Activation, DNA Repair, and Cell Survival through Ablation of β-TrCP2 in Response to Genotoxic Stress. *J. Biol. Chem.* **2021**, *296*, 100511, doi:10.1016/j.jbc.2021.100511.

15. zhang, Q.; wang, W.; gao, Q. β-TRCP-Mediated AEBP2 Ubiquitination and Destruction Controls Cisplatin Resistance in Ovarian Cancer. *Biochem Bioph Res Co* **2020**, *523*, 274–279, doi:10.1016/j.bbrc.2019.12.050.

16. Kim, T.Y.; Siesser, P.F.; Rossman, K.L.; Goldfarb, D.; Mackinnon, K.; Yan, F.; Yi, X.; MacCoss, M.J.; Moon, R.T.; Der, C.J.; et al. Substrate Trapping Proteomics Reveals Targets of the BTrCP2/FBXW11 Ubiquitin Ligase. *Mol Cell Biol* **2015**, *35*, 167–181, doi:10.1128/mcb.00857-14.

17. Li, N.; Liu, Q.; Han, Y.; Pei, S.; Cheng, B.; Xu, J.; Miao, X.; Pan, Q.; Wang, H.; Guo, J.; et al. ARID1A Loss Induces Polymorphonuclear Myeloid-Derived Suppressor Cell Chemotaxis and Promotes Prostate Cancer Progression. *Nat. Commun.* **2022**, *13*, 7281, doi:10.1038/s41467-022-34871-9.

18. Jiang, Z.; Peng, T.; Qian, H.; Lu, C.; Qiu, F.; Zhang, S. DNA Damage-Induced Activation of ATM Promotes β-TRCP-Mediated ARID1A Ubiquitination and Destruction in Gastric Cancer Cells. *Cancer Cell Int* **2019**, *19*, 162, doi:10.1186/s12935-019-0878-y.

19. Frank, C.L.; Ge, X.; Xie, Z.; Zhou, Y.; Tsai, L.-H. Control of Activating Transcription Factor 4 (ATF4) Persistence by Multisite Phosphorylation Impacts Cell Cycle Progression and Neurogenesis*. *J Biol Chem* **2010**, *285*, 33324–33337, doi:10.1074/jbc.m110.140699.

20. Lassot, I.; Ségéral, E.; Berlioz-Torrent, C.; Durand, H.; Groussin, L.; Hai, T.; Benarous, R.; Margottin-Goguet, F. ATF4 Degradation Relies on a Phosphorylation-Dependent Interaction with the SCFβTrCPUbiquitin Ligase. *Mol Cell Biol* **2001**, *21*, 2192–2202, doi:10.1128/mcb.21.6.2192-2202.2001.

21. Li, M.-L.; Defren, J.; Brewer, G. Hsp27 and F-Box Protein β-TrCP Promote Degradation of MRNA Decay Factor AUF1. *Mol. Cell. Biol.* **2013**, *33*, 2315–2326, doi:10.1128/mcb.00931-12.

22. Lobry, C.; Lopez, T.; Israël, A.; Weil, R. Negative Feedback Loop in T Cell Activation through IκB Kinase-Induced Phosphorylation and Degradation of Bcl10. *Proc National Acad Sci* **2007**, *104*, 908–913, doi:10.1073/pnas.0606982104.

23. Dehan, E.; Bassermann, F.; Guardavaccaro, D.; Vasiliver-Shamis, G.; Cohen, M.; Lowes, K.N.; Dustin, M.; Huang, D.C.S.; Taunton, J.; Pagano, M. BTrCP- and Rsk1/2-Mediated Degradation of BimEL Inhibits Apoptosis. *Mol Cell* **2009**, *33*, 109–116, doi:10.1016/j.molcel.2008.12.020.

24. Moustafa-Kamal, M.; Gamache, I.; Lu, Y.; Li, S.; Teodoro, J.G. BimEL Is Phosphorylated at Mitosis by Aurora A and Targeted for Degradation by BTrCP1. *Cell Death Differ.* **2013**, *20*, 1393–1403, doi:10.1038/cdd.2013.93.

25. Sahasrabuddhe, A.A.; Dimri, M.; Bommi, P.V.; Dimri, G.P. BTrCP Regulates BMI1 Protein Turnover via Ubiquitination and Degradation. *Cell Cycle* **2011**, *10*, 1322–1330, doi:10.4161/cc.10.8.15372.

26. Seki, A.; Coppinger, J.A.; Du, H.; Jang, C.-Y.; Yates, J.R.; Fang, G. Plk1- and β-TrCP–Dependent Degradation of Bora Controls Mitotic Progression. *J Cell Biology* **2008**, *181*, 65–78, doi:10.1083/jcb.200712027.

27. Chan, E.H.Y.; Santamaria, A.; Silljé, H.H.W.; Nigg, E.A. Plk1 Regulates Mitotic Aurora A Function through BTrCP-Dependent Degradation of HBora. *Chromosoma* **2008**, *117*, 457–469, doi:10.1007/s00412-008-0165-5.

28. Qin, B.; Gao, B.; Yu, J.; Yuan, J.; Lou, Z. Ataxia Telangiectasia-Mutated- and Rad3-Related Protein Regulates the DNA Damage-Induced G2/M Checkpoint through the Aurora A Cofactor Bora Protein*. *J Biol Chem* **2013**, *288*, 16139–16144, doi:10.1074/jbc.m113.456780.

29. Chen, Y.; Chang, L. Gallic Acid Downregulates Matrix Metalloproteinase-2 (MMP-2) and MMP-9 in Human Leukemia Cells with Expressed Bcr/Abl. *Mol. Nutr. Food Res.* **2012**, *56*, 1398–1412, doi:10.1002/mnfr.201200167.

30. Sasajima, H.; Nakagawa, K.; Kashiwayanagi, M.; Yokosawa, H. Polyubiquitination of the B-Cell Translocation Gene 1 and 2 Proteins Is Promoted by the SCF Ubiquitin Ligase Complex Containing BTrCP. *Biological Pharm Bulletin* **2012**, *35*, 1539–1545, doi:10.1248/bpb.b12-00330.

31. Tan, M.; Gallegos, J.R.; Gu, Q.; Huang, Y.; Li, J.; Jin, Y.; Lu, H.; Sun, Y. SAG/ROC-SCFβ-TrCP E3 Ubiquitin Ligase Promotes Pro-Caspase-3 Degradation as a Mechanism of Apoptosis Protection. *Neoplasia* **2006**, *8*, 1042–1054, doi:10.1593/neo.06568.

32. Wang, N.; Wang, X.; Tan, H.-Y.; Li, S.; Tsang, C.M.; Tsao, S.-W.; Feng, Y. Berberine Suppresses Cyclin D1 Expression through Proteasomal Degradation in Human Hepatoma Cells. *Int J Mol Sci* **2016**, *17*, 1899, doi:10.3390/ijms17111899.

33. Mavrommati, I.; Faedda, R.; Galasso, G.; Li, J.; Burdova, K.; Fischer, R.; Kessler, B.M.; Carrero, Z.I.; Guardavaccaro, D.; Pagano, M.; et al. β-TrCP- and Casein Kinase II-Mediated Degradation of Cyclin F Controls Timely Mitotic Progression. *Cell Reports* **2018**, *24*, 3404–3412, doi:10.1016/j.celrep.2018.08.076.

34. Bour, S.; Geleziunas, R.; Wainberg, M.A. The Human Immunodeficiency Virus Type 1 (HIV-1) CD4 Receptor and Its Central Role in Promotion of HIV-1 Infection. *Microbiol Rev* **1995**, *59*, 63–93, doi:10.1128/mr.59.1.63-93.1995.

35. Margottin, F.; Bour, S.P.; Durand, H.; Selig, L.; Benichou, S.; Richard, V.; Thomas, D.; Strebel, K.; Benarous, R. A Novel Human WD Protein, h-BTrCP, That Interacts with HIV-1 Vpu Connects CD4 to the ER Degradation Pathway through an F-Box Motif. *Mol Cell* **1998**, *1*, 565–574, doi:10.1016/s1097-2765(00)80056-8.

36. Fukushima, H.; Ogura, K.; Wan, L.; Lu, Y.; Li, V.; Gao, D.; Liu, P.; Lau, A.W.; Wu, T.; Kirschner, M.W.; et al. SCF-Mediated Cdh1 Degradation Defines a Negative Feedback System That Coordinates Cell-Cycle Progression. *Cell Rep.* **2013**, *4*, 803–816, doi:10.1016/j.celrep.2013.07.031.

37. Kang, T.; Wei, Y.; Honaker, Y.; Yamaguchi, H.; Appella, E.; Hung, M.-C.; Piwnica-Worms, H. GSK-3β Targets Cdc25A for Ubiquitin-Mediated Proteolysis, and GSK-3β Inactivation Correlates with Cdc25A Overproduction in Human Cancers. *Cancer Cell* **2008**, *13*, 36–47, doi:10.1016/j.ccr.2007.12.002.

38. Honaker, Y.; Piwnica-Worms, H. Casein Kinase 1 Functions as Both Penultimate and Ultimate Kinase in Regulating Cdc25A Destruction. *Oncogene* **2010**, *29*, 3324–3334, doi:10.1038/onc.2010.96.

39. Busino, L.; Donzelli, M.; Chiesa, M.; Guardavaccaro, D.; Ganoth, D.; Dorrello, N.V.; Hershko, A.; Pagano, M.; Draetta, G.F. Degradation of Cdc25A by β-TrCP during S Phase and in Response to DNA Damage. *Nature* **2003**, *426*, 87–91, doi:10.1038/nature02082.

40. Jin, J.; Shirogane, T.; Xu, L.; Nalepa, G.; Qin, J.; Elledge, S.J.; Harper, J.W. SCFβ-TRCP Links Chk1 Signaling to Degradation of the Cdc25A Protein Phosphatase. *Gene Dev* **2003**, *17*, 3062–3074, doi:10.1101/gad.1157503.

41. Melixetian, M.; Klein, D.K.; Sørensen, C.S.; Helin, K. NEK11 Regulates CDC25A Degradation and the IR-Induced G2/M Checkpoint. *Nat Cell Biol* **2009**, *11*, 1247–1253, doi:10.1038/ncb1969.

42. Piao, S.; Lee, S.-J.; Xu, Y.; Gwak, J.; Oh, S.; Park, B.-J.; Ha, N.-C. CK1ε Targets Cdc25A for Ubiquitin-Mediated Proteolysis under Normal Conditions and in Response to Checkpoint Activation. *Cell Cycle* **2011**, *10*, 531–537, doi:10.4161/cc.10.3.14757.

43. Uchida, S.; Yoshioka, K.; Kizu, R.; Nakagama, H.; Matsunaga, T.; Ishizaka, Y.; Poon, R.Y.C.; Yamashita, K. Stress-Activated Mitogen-Activated Protein Kinases c-Jun NH2-Terminal Kinase and P38 Target Cdc25B for Degradation. *Cancer Res* **2009**, *69*, 6438–6444, doi:10.1158/0008-5472.can-09-0869.

44. Uchida, S.; Watanabe, N.; Kudo, Y.; Yoshioka, K.; Matsunaga, T.; Ishizaka, Y.; Nakagama, H.; Poon, R.Y.C.; Yamashita, K. SCFβ(TrCP) Mediates Stress-Activated MAPK-Induced Cdc25B Degradation. *J Cell Sci* **2011**, *124*, 2816–2825, doi:10.1242/jcs.083931.

45. Kanemori, Y.; Uto, K.; Sagata, N. β-TrCP Recognizes a Previously Undescribed Nonphosphorylated Destruction Motif in Cdc25A and Cdc25B Phosphatases. *P Natl Acad Sci Usa* **2005**, *102*, 6279–6284, doi:10.1073/pnas.0501873102.

46. Thomas, Y.; Coux, O.; Baldin, V. BTrCP-Dependent Degradation of CDC25B Phosphatase at the Metaphase-Anaphase Transition Is a Pre-Requisite for Correct Mitotic Exit. *Cell Cycle* **2010**, *9*, 4338–4350, doi:10.4161/cc.9.21.13593.

47. Nakagawa, T.; Araki, T.; Nakagawa, M.; Hirao, A.; Unno, M.; Nakayama, K. S6 Kinase- and β-TrCP2-Dependent Degradation of P19Arf Is Required for Cell Proliferation. *Mol. Cell. Biol.* **2015**, *35*, 3517–3527, doi:10.1128/mcb.00343-15.

48. Liu, G.; Zhang, Q.; Xia, L.; Shi, M.; Cai, J.; Zhang, H.; Li, J.; Lin, G.; Xie, W.; Zhang, Y.; et al. RNA-Binding Protein CELF6 Is Cell Cycle Regulated and Controls Cancer Cell Proliferation by Stabilizing P21. *Cell Death Dis* **2019**, *10*, 688, doi:10.1038/s41419-019-1927-0.

49. Pagan, J.K.; Marzio, A.; Jones, M.J.K.; Saraf, A.; Jallepalli, P.V.; Florens, L.; Washburn, M.P.; Pagano, M. Degradation of Cep68 and PCNT Cleavage Mediate Cep215 Removal from the PCM to Allow Centriole Separation, Disengagement and Licensing. *Nat Cell Biol* **2015**, *17*, 31–43, doi:10.1038/ncb3076.

50. Man, X.; Megraw, T.L.; Lim, Y.P. Cep68 Can Be Regulated by Nek2 and SCF Complex. *Eur. J. Cell Biol.* **2015**, *94*, 162–172, doi:10.1016/j.ejcb.2015.01.004.

51. Zhao, D.; Lu, X.; Wang, G.; Lan, Z.; Liao, W.; Li, J.; Liang, X.; Chen, J.R.; Shah, S.; Shang, X.; et al. Synthetic Essentiality of Chromatin Remodelling Factor CHD1 in PTEN-Deficient Cancer. *Nature* **2017**, *542*, 484–488, doi:10.1038/nature21357.

52. Ma, Y.; Cui, D.; Wang, L.; Wang, Y.; Yang, F.; Pan, H.; Gong, L.; Zhang, M.; Xiong, X.; Zhao, Y. P90 Ribosomal S6 Kinase Confers Cancer Cell Survival by Mediating Checkpoint Kinase 1 Degradation in Response to Glucose Stress. *Cancer Sci.* **2022**, *113*, 132–144, doi:10.1111/cas.15168.

53. Ma, Y.; Cui, D.; Xiong, X.; Inuzuka, H.; Wei, W.; Sun, Y.; North, B.J.; Zhao, Y. SCFβ-TrCP Ubiquitinates CHK1 in an AMPK-dependent Manner in Response to Glucose Deprivation. *Mol Oncol* **2019**, *13*, 307–321, doi:10.1002/1878-0261.12403.

54. Park, S.; Oh, A.-Y.; Cho, J.-H.; Yoon, M.-H.; Woo, T.-G.; Kang, S.; Lee, H.-Y.; Jung, Y.; Park, B.-J. Therapeutic Effect of Quinacrine, an Anti-Protozoan Drug, by Selective Suppression of p-CHK1/2 in P53-Negative Malignant Cancers. *Mol Cancer Res* **2018**, *16*, molcanres.0511.2017, doi:10.1158/1541-7786.mcr-17-0511.

55. Mamely, I.; Vugt, M.A. van; Smits, V.A.; Semple, J.I.; Lemmens, B.; Perrakis, A.; Medema, R.H.; Freire, R. Polo-like Kinase-1 Controls Proteasome-Dependent Degradation of Claspin during Checkpoint Recovery. *Curr Biol* **2006**, *16*, 1950–1955, doi:10.1016/j.cub.2006.08.026.

56. Cabrera, E.; Raninga, P.; Khanna, K.K.; Freire, R. GSK3-β Stimulates Claspin Degradation via β-TrCP Ubiquitin Ligase and Alters Cancer Cell Survival. *Cancers* **2019**, *11*, 1073, doi:10.3390/cancers11081073.

57. Zhao, J.; Wei, J.; Mialki, R.; Zou, C.; Mallampalli, R.K.; Zhao, Y. Extracellular Signal-Regulated Kinase (ERK) Regulates Cortactin Ubiquitination and Degradation in Lung Epithelial Cells*. *J. Biol. Chem.* **2012**, *287*, 19105–19114, doi:10.1074/jbc.m112.339507.

58. Cheng, Y.; Gao, W.-W.; Tang, H.-M.V.; Deng, J.-J.; Wong, C.-M.; Chan, C.-P.; Jin, D.-Y. β-TrCP-Mediated Ubiquitination and Degradation of Liver-Enriched Transcription Factor CREB-H. *Sci. Rep.* **2016**, *6*, 23938, doi:10.1038/srep23938.

59. Shrestha, H.; Yuan, T.; He, Y.; Moon, P.-G.; Shrestha, N.; Ryu, T.; Park, S.-Y.; Cho, Y.-C.; Lee, C.-H.; Baek, M.-C.; et al. Investigation of the Molecular Mechanism of δ-Catenin Ubiquitination: Implication of β-TrCP-1 as a Potential E3 Ligase. *Biochim. Biophys. Acta (BBA) - Mol. Cell Res.* **2016**, *1863*, 2311–2321, doi:10.1016/j.bbamcr.2016.06.006.

60. Wu, X.; Fukushima, H.; North, B.J.; Nagaoka, Y.; Nagashima, K.; Deng, F.; Okabe, K.; Inuzuka, H.; Wei, W. SCFβ-TRCP Regulates Osteoclastogenesis via Promoting CYLD Ubiquitination. *Oncotarget* **2014**, *5*, 4211–4221, doi:10.18632/oncotarget.1971.

61. Katiyar, S.; Liu, E.; Knutzen, C.A.; Lang, E.S.; Lombardo, C.R.; Sankar, S.; Toth, J.I.; Petroski, M.D.; Ronai, Z.; Chiang, G.G. REDD1, an Inhibitor of MTOR Signalling, Is Regulated by the CUL4A–DDB1 Ubiquitin Ligase. *Embo Rep* **2009**, *10*, 866–872, doi:10.1038/embor.2009.93.

62. Kim, J.; D'Annibale, S.; Magliozzi, R.; Low, T.Y.; Jansen, P.; Shaltiel, I.A.; Mohammed, S.; Heck, A.J.R.; Medema, R.H.; Guardavaccaro, D. USP17- and SCFβTrCP-Regulated Degradation of DEC1 Controls the DNA Damage Response. *Mol. Cell. Biol.* **2014**, *34*, 4177–4185, doi:10.1128/mcb.00530-14.

63. Teh, A.-H.; Yeap, K.-H.; Hisano, T. Insights into DEPTOR Regulation from in Silico Analysis of DEPTOR Complexes. *J. Struct. Biol.* **2020**, *212*, 107602, doi:10.1016/j.jsb.2020.107602.

64. Gao, D.; Inuzuka, H.; Tan, M.-K.M.; Fukushima, H.; Locasale, J.W.; Liu, P.; Wan, L.; Zhai, B.; Chin, Y.R.; Shaik, S.; et al. MTOR Drives Its Own Activation via SCFβTrCP-Dependent Degradation of the MTOR Inhibitor DEPTOR. *Mol Cell* **2011**, *44*, 290–303, doi:10.1016/j.molcel.2011.08.030.

65. Duan, S.; Skaar, J.R.; Kuchay, S.; Toschi, A.; Kanarek, N.; Ben-Neriah, Y.; Pagano, M. MTOR Generates an Auto-Amplification Loop by Triggering the BTrCP- and CK1α-Dependent Degradation of DEPTOR. *Mol Cell* **2011**, *44*, 317–324, doi:10.1016/j.molcel.2011.09.005.

66. Zhao, Y.; Xiong, X.; Sun, Y. DEPTOR, an MTOR Inhibitor, Is a Physiological Substrate of SCFβTrCP E3 Ubiquitin Ligase and Regulates Survival and Autophagy. *Mol Cell* **2011**, *44*, 304–316, doi:10.1016/j.molcel.2011.08.029.

67. Mantovani, F.; Banks, L. Regulation of the Discs Large Tumor Suppressor by a Phosphorylation-Dependent Interaction with the β-TrCP Ubiquitin Ligase Receptor*. *J Biol Chem* **2003**, *278*, 42477–42486, doi:10.1074/jbc.m302799200.

68. Nakagawa, T.; Zhang, T.; Kushi, R.; Nakano, S.; Endo, T.; Nakagawa, M.; Yanagihara, N.; Zarkower, D.; Nakayama, K. Regulation of Mitosis-Meiosis Transition by the Ubiquitin Ligase β-TrCP in Male Germ Cells. *Development* **2017**, *144*, dev.158485, doi:10.1242/dev.158485.

69. FANG, Q.-L.; YIN, Y.-R.; XIE, C.-R.; ZHANG, S.; ZHAO, W.-X.; PAN, C.; WANG, X.-M.; YIN, Z.-Y. Mechanistic and Biological Significance of DNA Methyltransferase 1 Upregulated by Growth Factors in Human Hepatocellular Carcinoma. *Int. J. Oncol.* **2014**, *46*, 782–790, doi:10.3892/ijo.2014.2776.

70. Chu, P.-C.; Chuang, H.-C.; Kulp, S.K.; Chen, C.-S. The MRNA-Stabilizing Factor HuR Protein Is Targeted by β-TrCP Protein for Degradation in Response to Glycolysis Inhibition*. *J. Biol. Chem.* **2012**, *287*, 43639–43650, doi:10.1074/jbc.m112.393678.

71. Manavathi, B.; Rayala, S.K.; Kumar, R. Phosphorylation-Dependent Regulation of Stability and Transforming Potential of ETS Transcriptional Factor ESE-1 by P21-Activated Kinase 1*. *J Biol Chem* **2007**, *282*, 19820–19830, doi:10.1074/jbc.m702309200.

72. Xie, C.-M.; Wei, D.; Zhao, L.; Marchetto, S.; Mei, L.; Borg, J.-P.; Sun, Y. Erbin Is a Novel Substrate of the Sag-BTrCP E3 Ligase That Regulates KrasG12D-Induced Skin Tumorigenesis. *J. Cell Biol.* **2015**, *209*, 721–738, doi:10.1083/jcb.201411104.

73. Sahasrabuddhe, A.A.; Chen, X.; Chung, F.; Velusamy, T.; Lim, M.S.; Elenitoba-Johnson, K.S.J. Oncogenic Y641 Mutations in EZH2 Prevent Jak2/β-TrCP-Mediated Degradation. *Oncogene* **2015**, *34*, 445–454, doi:10.1038/onc.2013.571.

74. Kee, Y.; Kim, J.M.; D'Andrea, A.D.; D'Andrea, A. Regulated Degradation of FANCM in the Fanconi Anemia Pathway during Mitosis. *Gene Dev* **2009**, *23*, 555–560, doi:10.1101/gad.1761309.

75. Guardavaccaro, D.; Kudo, Y.; Boulaire, J.; Barchi, M.; Busino, L.; Donzelli, M.; Margottin-Goguet, F.; Jackson, P.K.; Yamasaki, L.; Pagano, M. Control of Meiotic and Mitotic Progression by the F Box Protein β-Trcp1 In Vivo. *Dev Cell* **2003**, *4*, 799–812, doi:10.1016/s1534-5807(03)00154-0.

76. Margottin-Goguet, F.; Hsu, J.Y.; Loktev, A.; Hsieh, H.-M.; Reimann, J.D.R.; Jackson, P.K. Prophase Destruction of Emi1 by the SCFβTrCP/Slimb Ubiquitin Ligase Activates the Anaphase Promoting Complex to Allow Progression beyond Prometaphase. *Dev Cell* **2003**, *4*, 813–826, doi:10.1016/s1534-5807(03)00153-9.

77. Moshe, Y.; Boulaire, J.; Pagano, M.; Hershko, A. Role of Polo-like Kinase in the Degradation of Early Mitotic Inhibitor 1, a Regulator of the Anaphase Promoting Complex/Cyclosome. *P Natl Acad Sci Usa* **2004**, *101*, 7937–7942, doi:10.1073/pnas.0402442101.

78. Xu, J.; Zhou, W.; Yang, F.; Chen, G.; Li, H.; Zhao, Y.; Liu, P.; Li, H.; Tan, M.; Xiong, X.; et al. The β-TrCP-FBXW2-SKP2 Axis Regulates Lung Cancer Cell Growth with FBXW2 Acting as a Tumour Suppressor. *Nat Commun* **2017**, *8*, 14002, doi:10.1038/ncomms14002.

79. Ray, D.; Osmundson, E.C.; Kiyokawa, H. Constitutive and UV-Induced Fibronectin Degradation Is a Ubiquitination-Dependent Process Controlled by β-TrCP*. *J Biol Chem* **2006**, *281*, 23060–23065, doi:10.1074/jbc.m604311200.

80. Hayakawa, M.; Kitagawa, H.; Miyazawa, K.; Kitagawa, M.; Kikugawa, K. The FWD1/B-TrCP-mediated Degradation Pathway Establishes a 'Turning off Switch' of a Cdc42 Guanine Nucleotide Exchange Factor, FGD1. *Genes Cells* **2005**, *10*, 241–251, doi:10.1111/j.1365-2443.2005.00834.x.

81. Hayakawa, M.; Matsushima, M.; Hagiwara, H.; Oshima, T.; Fujino, T.; Ando, K.; Kikugawa, K.; Tanaka, H.; Miyazawa, K.; Kitagawa, M. Novel Insights into FGD3, a Putative GEF for Cdc42, That Undergoes SCFFWD1/B-TrCP-mediated Proteasomal Degradation Analogous to That of Its Homologue FGD1 but Regulates Cell Morphology and Motility Differently from FGD1. *Genes Cells* **2008**, *13*, 329–342, doi:10.1111/j.1365-2443.2008.01168.x.

82. Nagashima, K.; Fukushima, H.; Shimizu, K.; Yamada, A.; Hidaka, M.; Hasumi, H.; Ikebe, T.; Fukumoto, S.; Okabe, K.; Inuzuka, H. Nutrient-Induced FNIP Degradation by SCFβ-TRCP Regulates FLCN Complex Localization and Promotes Renal Cancer Progression. *Oncotarget* **2016**, *8*, 9947–9960, doi:10.18632/oncotarget.14221.

83. Ma, J.; Lu, Y.; Zhang, S.; Li, Y.; Huang, J.; Yin, Z.; Ren, J.; Huang, K.; Liu, L.; Yang, K.; et al. β-Trcp Ubiquitin Ligase and RSK2 Kinase-Mediated Degradation of FOXN2 Promotes Tumorigenesis and Radioresistance in Lung Cancer. *Cell Death Differ* **2018**, *25*, 1473–1485, doi:10.1038/s41418-017-0055-6.

84. Tsai, W.-B.; Chung, Y.M.; Zou, Y.; Park, S.-H.; Xu, Z.; Nakayama, K.; Lin, S.-H.; Hu, M.C.-T. Inhibition of FOXO3 Tumor Suppressor Function by BTrCP1 through Ubiquitin-Mediated Degradation in a Tumor Mouse Model. *Plos One* **2010**, *5*, e11171, doi:10.1371/journal.pone.0011171.

85. Su, J.-L.; Cheng, X.; Yamaguchi, H.; Chang, Y.-W.; Hou, C.-F.; Lee, D.-F.; Ko, H.-W.; Hua, K.-T.; Wang, Y.-N.; Hsiao, M.; et al. FOXO3a-Dependent Mechanism of E1A-Induced Chemosensitization. *Cancer Res* **2011**, *71*, 6878–6887, doi:10.1158/0008-5472.can-11-0295.

86. Wang, S.; Zhang, Y.; Wang, Y.; Ye, P.; Li, J.; Li, H.; Ding, Q.; Xia, J. Amphiregulin Confers Regulatory T Cell Suppressive Function and Tumor Invasion via the EGFR/GSK-3β/Foxp3 Axis*. *J. Biol. Chem.* **2016**, *291*, 21085–21095, doi:10.1074/jbc.m116.717892.

87. Magliozzi, R.; Carrero, Z.I.; Low, T.Y.; Yuniati, L.; Valdes-Quezada, C.; Kruiswijk, F.; Wijk, K. van; Heck, A.J.R.; Jackson, C.L.; Guardavaccaro, D. Inheritance of the Golgi Apparatus and Cytokinesis Are Controlled by Degradation of GBF1. *Cell Rep.* **2018**, *23*, 3381-3391.e4, doi:10.1016/j.celrep.2018.05.031.

88. Kerkhof, P. van; Putters, J.; Strous, G.J. The Ubiquitin Ligase SCF(BTrCP) Regulates the Degradation of the Growth Hormone Receptor*. *J Biol Chem* **2007**, *282*, 20475–20483, doi:10.1074/jbc.m702610200.

89. Zhang, R.; Huang, S.Y.; Li, K.K.-W.; Li, Y.-H.; Hsu, W.-H.; Zhang, G.; Chang, C.-J.; Yang, J.-Y. Dual Degradation Signals Destruct GLI1: AMPK Inhibits GLI1 through β-TrCP-Mediated Proteasome Degradation. *Oncotarget* **2014**, *5*, doi:10.18632/oncotarget.17769.

90. Bhatia, N.; Thiyagarajan, S.; Elcheva, I.; Saleem, M.; Dlugosz, A.; Mukhtar, H.; Spiegelman, V.S. Gli2 Is Targeted for Ubiquitination and Degradation by β-TrCP Ubiquitin Ligase. *J Biol Chem* **2006**, *281*, 19320–19326, doi:10.1074/jbc.m513203200.

91. Gao, C.; Chen, G.; Romero, G.; Moschos, S.; Xu, X.; Hu, J. Induction of Gsk3β-β-TrCP Interaction Is Required for Late Phase Stabilization of β-Catenin in Canonical Wnt Signaling*. *J Biol Chem* **2014**, *289*, 7099–7108, doi:10.1074/jbc.m113.532606.

92. Wang, H.-M.; Xu, Y.-F.; Ning, S.-L.; Yang, D.-X.; Li, Y.; Du, Y.-J.; Yang, F.; Zhang, Y.; Liang, N.; Yao, W.; et al. The Catalytic Region and PEST Domain of PTPN18 Distinctly Regulate the HER2 Phosphorylation and Ubiquitination Barcodes. *Cell Res* **2014**, *24*, 1067–1090, doi:10.1038/cr.2014.99.

93. Bi, Y.; Chen, X.; Wei, B.; Wang, L.; Gong, L.; Li, H.; Xiong, X.; Zhao, Y. DEPTOR Stabilizes ErbB2 to Promote the Proliferation and Survival of ErbB2-Positive Breast Cancer Cells. *Theranostics* **2021**, *11*, 6355–6369, doi:10.7150/thno.51286.

94. Yao, J.; Yang, J.; Yang, Z.; Wang, X.-P.; Yang, T.; Ji, B.; Zhang, Z.-Y. FBXW11 Contributes to Stem-Cell-like Features and Liver Metastasis through Regulating HIC1-Mediated SIRT1 Transcription in Colorectal Cancer. *Cell Death Dis.* **2021**, *12*, 930, doi:10.1038/s41419-021-04185-7.

95. Mennerich, D.; Kubaichuk, K.; Raza, G.S.; Fuhrmann, D.C.; Herzig, K.-H.; Brüne, B.; Kietzmann, T. ER-Stress Promotes VHL-Independent Degradation of Hypoxia-Inducible Factors via FBXW1A/BTrCP. *Redox Biol.* **2022**, *50*, 102243, doi:10.1016/j.redox.2022.102243.

96. Jeong, W.-J.; Yoon, J.; Park, J.-C.; Lee, S.-H.; Lee, S.-H.; Kaduwal, S.; Kim, H.; Yoon, J.-B.; Choi, K.-Y. Ras Stabilization Through Aberrant Activation of Wnt/β-Catenin Signaling Promotes Intestinal Tumorigenesis. *Sci Signal* **2012**, *5*, ra30, doi:10.1126/scisignal.2002242.

97. Lee, Y.-J.; Kim, E.-H.; Lee, J.S.; Jeoung, D.; Bae, S.; Kwon, S.H.; Lee, Y.-S. HSF1 as a Mitotic Regulator: Phosphorylation of HSF1 by Plk1 Is Essential for Mitotic Progression. *Cancer Res* **2008**, *68*, 7550–7560, doi:10.1158/0008-5472.can-08-0129.

98. Bhattacharya, S.; HuangFu, W.-C.; Liu, J.; Veeranki, S.; Baker, D.P.; Koumenis, C.; Diehl, J.A.; Fuchs, S.Y. Inducible Priming Phosphorylation Promotes Ligand-Independent Degradation of the IFNAR1 Chain of Type I Interferon Receptor*. *J Biol Chem* **2010**, *285*, 2318–2325, doi:10.1074/jbc.m109.071498.

99. Kumar, K.G.S.; Tang, W.; Ravindranath, A.K.; Clark, W.A.; Croze, E.; Fuchs, S.Y. SCFHOS Ubiquitin Ligase Mediates the Ligand-induced Down-regulation of the Interferon-α Receptor. *Embo J* **2003**, *22*, 5480–5490, doi:10.1093/emboj/cdg524.

100. Kumar, K.G.S.; Krolewski, J.J.; Fuchs, S.Y. Phosphorylation and Specific Ubiquitin Acceptor Sites Are Required for Ubiquitination and Degradation of the IFNAR1 Subunit of Type I Interferon Receptor. *J Biol Chem* **2004**, *279*, 46614–46620, doi:10.1074/jbc.m407082200.

101. Chen, Z.; Hagler, J.; Palombella, V.J.; Melandri, F.; Scherer, D.; Ballard, D.; Maniatis, T. Signal-Induced Site-Specific Phosphorylation Targets I Kappa B Alpha to the Ubiquitin-Proteasome Pathway. *Gene Dev* **1995**, *9*, 1586–1597, doi:10.1101/gad.9.13.1586.

102. Alkalay, I.; Yaron, A.; Hatzubai, A.; Orian, A.; Ciechanover, A.; Ben-Neriah, Y. Stimulation-Dependent I Kappa B Alpha Phosphorylation Marks the NF-Kappa B Inhibitor for Degradation via the Ubiquitin-Proteasome Pathway. *Proc National Acad Sci* **1995**, *92*, 10599–10603, doi:10.1073/pnas.92.23.10599.

103. Spencer, E.; Jiang, J.; Chen, Z.J. Signal-Induced Ubiquitination of IκBα by the F-Box Protein Slimb/β-TrCP. *Gene Dev* **1999**, *13*, 284–294, doi:10.1101/gad.13.3.284.

104. Hatakeyama, S.; Kitagawa, M.; Nakayama, K.; Shirane, M.; Matsumoto, M.; Hattori, K.; Higashi, H.; Nakano, H.; Okumura, K.; Onoé, K.; et al. Ubiquitin-Dependent Degradation of IκBα Is Mediated by a Ubiquitin Ligase Skp1/Cul 1/F-Box Protein FWD1. *Proc National Acad Sci* **1999**, *96*, 3859–3863, doi:10.1073/pnas.96.7.3859.

105. Kroll, M.; Margottin, F.; Kohl, A.; Renard, P.; Durand, H.; Concordet, J.-P.; Bachelerie, F.; Arenzana-Seisdedos, F.; Benarous, R. Inducible Degradation of IκBα by the Proteasome Requires Interaction with the F-Box Protein h-BTrCP*. *J Biol Chem* **1999**, *274*, 7941–7945, doi:10.1074/jbc.274.12.7941.

106. Suzuki, H.; Chiba, T.; Kobayashi, M.; Takeuchi, M.; Suzuki, T.; Ichiyama, A.; Ikenoue, T.; Omata, M.; Furuichi, K.; Tanaka, K. IκBα Ubiquitination Is Catalyzed by an SCF-like Complex Containing Skp1, Cullin-1, and Two F-Box/WD40-Repeat Proteins, BTrCP1 and BTrCP2. *Biochem Bioph Res Co* **1999**, *256*, 127–132, doi:10.1006/bbrc.1999.0289.

107. Yaron, A.; Hatzubai, A.; Davis, M.; Lavon, I.; Amit, S.; Manning, A.M.; Andersen, J.S.; Mann, M.; Mercurio, F.; Ben-Neriah, Y. Identification of the Receptor Component of the IκBα–Ubiquitin Ligase. *Nature* **1998**, *396*, 590–594, doi:10.1038/25159.

108. Tsuchiya, Y.; Asano, T.; Nakayama, K.; Kato, T.; Karin, M.; Kamata, H. Nuclear IKKbeta Is an Adaptor Protein for IkappaBalpha Ubiquitination and Degradation in UV-Induced NF-KappaB Activation. *Mol Cell* **2009**, *39*, 570–582, doi:10.1016/j.molcel.2010.07.030.

109. Soldatenkov, V.A.; Dritschilo, A.; Ronai, Z.; Fuchs, S.Y. Inhibition of Homologue of Slimb (HOS) Function Sensitizes Human Melanoma Cells for Apoptosis. *Cancer Res* **1999**, *59*, 5085–5088.

110. Shirane, M.; Hatakeyama, S.; Hattori, K.; Nakayama, K.; Nakayama, K. Common Pathway for the Ubiquitination of IκBα, IκBβ, and IκBε Mediated by the F-Box Protein FWD1*. *J Biol Chem* **1999**, *274*, 28169–28174, doi:10.1074/jbc.274.40.28169.

111. Wu, C.; Ghosh, S. β-TrCP Mediates the Signal-Induced Ubiquitination of IκBβ. *J Biol Chem* **1999**, *274*, 29591–29594, doi:10.1074/jbc.274.42.29591.

112. Jiang, H.; Lu, Y.; Yuan, L.; Liu, J. Regulation of Interleukin-10 Receptor Ubiquitination and Stability by Beta-TrCP-Containing Ubiquitin E3 Ligase. *Plos One* **2011**, *6*, e27464, doi:10.1371/journal.pone.0027464.

113. Cui, W.; Xiao, N.; Xiao, H.; Zhou, H.; Yu, M.; Gu, J.; Li, X. β-TrCP-Mediated IRAK1 Degradation Releases TAK1-TRAF6 from the Membrane to the Cytosol for TAK1-Dependent NF-KB Activation. *Mol. Cell. Biol.* **2012**, *32*, 3990–4000, doi:10.1128/mcb.00722-12.

114. Yoneyama, Y.; Inamitsu, T.; Chida, K.; Iemura, S.-I.; Natsume, T.; Maeda, T.; Hakuno, F.; Takahashi, S.-I. Serine Phosphorylation by MTORC1 Promotes IRS-1 Degradation through SCFβ-TRCP E3 Ubiquitin Ligase. *Iscience* **2018**, *5*, 1–18, doi:10.1016/j.isci.2018.06.006.

115. Wang, Z.; Dai, X.; Zhong, J.; Inuzuka, H.; Wan, L.; Li, X.; Wang, L.; Ye, X.; Sun, L.; Gao, D.; et al. SCFβ-TRCP Promotes Cell Growth by Targeting PR-Set7/Set8 for Degradation. *Nat Commun* **2015**, *6*, 10185, doi:10.1038/ncomms10185.

116. Zou, C.; Butler, P.L.; Coon, T.A.; Smith, R.M.; Hammen, G.; Zhao, Y.; Chen, B.B.; Mallampalli, R.K. LPS Impairs Phospholipid Synthesis by Triggering β-Transducin Repeat-Containing Protein (β-TrCP)-Mediated Polyubiquitination and Degradation of the Surfactant Enzyme Acyl-CoA:Lysophosphatidylcholine Acyltransferase I (LPCAT1)*. *J. Biological Chem.* **2011**, *286*, 2719–2727, doi:10.1074/jbc.m110.192377.

117. Shimizu, K.; Fukushima, H.; Ogura, K.; Lien, E.C.; Nihira, N.T.; Zhang, J.; North, B.J.; Guo, A.; Nagashima, K.; Nakagawa, T.; et al. The SCFβ-TRCP E3 Ubiquitin Ligase Complex Targets Lipin1 for Ubiquitination and Degradation to Promote Hepatic Lipogenesis. *Sci. Signal.* **2017**, *10*, doi:10.1126/scisignal.aah4117.

118. Lu, Y.; Li, X.; Liu, H.; Xue, J.; Zeng, Z.; Dong, X.; Zhang, T.; Wu, G.; Yang, K.; Xu, S. β-Trcp and CK1δ-Mediated Degradation of LZTS2 Activates PI3K/AKT Signaling to Drive Tumorigenesis and Metastasis in Hepatocellular Carcinoma. *Oncogene* **2021**, *40*, 1269–1283, doi:10.1038/s41388-020-01596-2.

119. Tang, X.; Chen, X.; Xu, Y.; Qiao, Y.; Zhang, X.; Wang, Y.; Guan, Y.; Sun, F.; Wang, J. CD166 Positively Regulates MCAM via Inhibition to Ubiquitin E3 Ligases Smurf1 and BTrCP through PI3K/AKT and c-Raf/MEK/ERK Signaling in Bel-7402 Hepatocellular Carcinoma Cells. *Cell. Signal.* **2015**, *27*, 1694–1702, doi:10.1016/j.cellsig.2015.05.006.

120. Ding, Q.; He, X.; Hsu, J.-M.; Xia, W.; Chen, C.-T.; Li, L.-Y.; Lee, D.-F.; Liu, J.-C.; Zhong, Q.; Wang, X.; et al. Degradation of Mcl-1 by β-TrCP Mediates Glycogen Synthase Kinase 3-Induced Tumor Suppression and Chemosensitization▿ †. *Mol Cell Biol* **2007**, *27*, 4006–4017, doi:10.1128/mcb.00620-06.

121. Ren, H.; Koo, J.; Guan, B.; Yue, P.; Deng, X.; Chen, M.; Khuri, F.R.; Sun, S.-Y. The E3 Ubiquitin Ligases β-TrCP and FBXW7 Cooperatively Mediates GSK3-Dependent Mcl-1 Degradation Induced by the Akt Inhibitor API-1, Resulting in Apoptosis. *Mol Cancer* **2013**, *12*, 146–146, doi:10.1186/1476-4598-12-146.

122. Westbrook, T.F.; Hu, G.; Ang, X.L.; Mulligan, P.; Pavlova, N.N.; Liang, A.; Leng, Y.; Maehr, R.; Shi, Y.; Harper, J.W.; et al. SCFβ-TRCP Controls Oncogenic Transformation and Neural Differentiation through REST Degradation. *Nature* **2008**, *452*, 370–374, doi:10.1038/nature06780.

123. Hu, X.; Liu, Z.; Duan, X.; Han, X.; Yuan, M.; Liu, L.; Xia, X.; Li, N.; Qin, J.; Wang, Y. Blocking MCT4 SUMOylation Inhibits the Growth of Breast Cancer Cells. *Mol. Carcinog.* **2021**, *60*, 702–714, doi:10.1002/mc.23336.

124. Inuzuka, H.; Tseng, A.; Gao, D.; Zhai, B.; Zhang, Q.; Shaik, S.; Wan, L.; Ang, X.L.; Mock, C.; Yin, H.; et al. Phosphorylation by Casein Kinase I Promotes the Turnover of the Mdm2 Oncoprotein via the SCFβ-TRCP Ubiquitin Ligase. *Cancer Cell* **2010**, *18*, 147–159, doi:10.1016/j.ccr.2010.06.015.

125. Lau, A.W.; Fukushima, H.; Wei, W. The Fbw7 and BetaTRCP E3 Ubiquitin Ligases and Their Roles in Tumorigenesis. *Front Biosci* **2012**, *17*, 2197, doi:10.2741/4045.

126. Schmidt, M.L.; Calvisi, D.F.; Clark, G.J. NORE1A Regulates MDM2 Via β-TrCP. *Cancers* **2016**, *8*, 39, doi:10.3390/cancers8040039.

127. Zhou, L.; Zhang, W.; Sun, Y.; Jia, L. Protein Neddylation and Its Alterations in Human Cancers for Targeted Therapy. *Cell. Signal.* **2018**, *44*, 92–102, doi:10.1016/j.cellsig.2018.01.009.

128. Kim, I.S.; Lee, M.; Park, J.H.; Jeon, R.; Baek, S.H.; Kim, K.I. BTrCP-Mediated Ubiquitylation Regulates Protein Stability of Mis18β in a Cell Cycle-Dependent Manner. *Biochem. Biophys. Res. Commun.* **2014**, *443*, 62–67, doi:10.1016/j.bbrc.2013.11.058.

129. Fiore, A.P.Z.P.; Rodrigues, A.M.; Ribeiro-Filho, H.V.; Manucci, A.C.; Ribeiro, P. de F.; Botelho, M.C.S.; Vogel, C.; Lopes-de-Oliveira, P.S.; Pagano, M.; Bruni-Cardoso, A. Extracellular Matrix Stiffness Regulates Degradation of MST2 via SCF BTrCP. *Biochim. Biophys. Acta (BBA) - Gen. Subj.* **2022**, *1866*, 130238, doi:10.1016/j.bbagen.2022.130238.

130. Zhong, J.; Shaik, S.; Wan, L.; Tron, A.E.; Wang, Z.; Sun, L.; Inuzuka, H.; Wei, W. SCF β-TRCP Targets MTSS1 for Ubiquitination-Mediated Destruction to Regulate Cancer Cell Proliferation and Migration. *Oncotarget* **2013**, *4*, 2339–2353, doi:10.18632/oncotarget.1446.

131. Huang, Y.; Hu, K.; Zhang, S.; Dong, X.; Yin, Z.; Meng, R.; Zhao, Y.; Dai, X.; Zhang, T.; Yang, K.; et al. S6K1 Phosphorylation-Dependent Degradation of Mxi1 by β-Trcp Ubiquitin Ligase Promotes Myc Activation and Radioresistance in Lung Cancer. *Theranostics* **2018**, *8*, 1286–1300, doi:10.7150/thno.22552.

132. Yang, X.; Zeng, Z.; Jie, X.; Wang, Y.; Han, J.; Zheng, Z.; Li, J.; Liu, H.; Dong, X.; Wu, G.; et al. Arginine Methyltransferase PRMT5 Methylates and Destabilizes Mxi1 to Confer Radioresistance in Non-Small Cell Lung Cancer. *Cancer Lett.* **2022**, *532*, 215594, doi:10.1016/j.canlet.2022.215594.

133. Liu, J.; Wan, L.; Liu, P.; Inuzuka, H.; Liu, J.; Wang, Z.; Wei, W. SCFβ-TRCP-Mediated Degradation of NEDD4 Inhibits Tumorigenesis through Modulating the PTEN/Akt Signaling Pathway. *Oncotarget* **2014**, *5*, 1026–1037, doi:10.18632/oncotarget.1675.

134. Chowdhury, A.M.M.A.; Katoh, H.; Hatanaka, A.; Iwanari, H.; Nakamura, N.; Hamakubo, T.; Natsume, T.; Waku, T.; Kobayashi, A. Multiple Regulatory Mechanisms of the Biological Function of NRF3 (NFE2L3) Control Cancer Cell Proliferation. *Sci Rep-uk* **2017**, *7*, 12494, doi:10.1038/s41598-017-12675-y.

135. Salmerón, A.; Janzen, J.; Soneji, Y.; Bump, N.; Kamens, J.; Allen, H.; Ley, S.C. Direct Phosphorylation of NF-KB1 P105 by the IκB Kinase Complex on Serine 927 Is Essential for Signal-Induced P105 Proteolysis*. *J Biol Chem* **2001**, *276*, 22215–22222, doi:10.1074/jbc.m101754200.

136. Lang, V.; Janzen, J.; Fischer, G.Z.; Soneji, Y.; Beinke, S.; Salmeron, A.; Allen, H.; Hay, R.T.; Ben-Neriah, Y.; Ley, S.C. BTrCP-Mediated Proteolysis of NF-KB1 P105 Requires Phosphorylation of P105 Serines 927 and 932. *Mol Cell Biol* **2003**, *23*, 402–413, doi:10.1128/mcb.23.1.402-413.2003.

137. Remouchamps, C.; Dejardin, E. Methods to Assess the Activation of the Alternative (Noncanonical) NF-KB Pathway by Non-Death TNF Receptors. *Methods Mol Biology* **2015**, *1280*, 103–119, doi:10.1007/978-1-4939-2422-6_7.

138. Liu, P.; Gan, W.; Guo, C.; Xie, A.; Gao, D.; Guo, J.; Zhang, J.; Willis, N.; Su, A.; Asara, J.M.; et al. Akt-Mediated Phosphorylation of XLF Impairs Non-Homologous End-Joining DNA Repair. *Mol Cell* **2015**, *57*, 648–661, doi:10.1016/j.molcel.2015.01.005.

139. Chowdhry, S.; Zhang, Y.; McMahon, M.; Sutherland, C.; Cuadrado, A.; Hayes, J.D. Nrf2 Is Controlled by Two Distinct β-TrCP Recognition Motifs in Its Neh6 Domain, One of Which Can Be Modulated by GSK-3 Activity. *Oncogene* **2013**, *32*, 3765–3781, doi:10.1038/onc.2012.388.

140. Rojo, A.I.; Rada, P.; Mendiola, M.; Ortega-Molina, A.; Wojdyla, K.; Rogowska-Wrzesinska, A.; Hardisson, D.; Serrano, M.; Cuadrado, A. The PTEN/NRF2 Axis Promotes Human Carcinogenesis. *Antioxid Redox Sign* **2014**, *21*, 2498–2514, doi:10.1089/ars.2014.5843.

141. Rada, P.; Rojo, A.I.; Chowdhry, S.; McMahon, M.; Hayes, J.D.; Cuadrado, A. SCF/β-TrCP Promotes Glycogen Synthase Kinase 3-Dependent Degradation of the Nrf2 Transcription Factor in a Keap1-Independent Manner. *Mol. Cell. Biol.* **2011**, *31*, 1121–1133, doi:10.1128/mcb.01204-10.

142. Mathur, A.; Pandey, V.K.; Kakkar, P. Activation of GSK3β/β-TrCP Axis via PHLPP1 Exacerbates Nrf2 Degradation Leading to Impairment in Cell Survival Pathway during Diabetic Nephropathy. *Free Radical Bio Med* **2018**, *120*, 414–424, doi:10.1016/j.freeradbiomed.2018.04.550.

143. Huang, K.-F.; Ma, K.-H.; Jhap, T.-Y.; Liu, P.-S.; Chueh, S.-H. Ultraviolet B Irradiation Induced Nrf2 Degradation Occurs via Activation of TRPV1 Channels in Human Dermal Fibroblasts. *Free Radical Bio Med* **2019**, *141*, 220–232, doi:10.1016/j.freeradbiomed.2019.06.020.

144. Dorrello, N.V.; Peschiaroli, A.; Guardavaccaro, D.; Colburn, N.H.; Sherman, N.E.; Pagano, M. S6K1- and SSTRCP-Mediated Degradation of PDCD4 Promotes Protein Translation and Cell Growth. *Science* **2006**, *314*, 467–471, doi:10.1126/science.1130276.

145. Matsuhashi, S.; Hamajima, H.; Xia, J.; Zhang, H.; Mizuta, T.; Anzai, K.; Ozaki, I. Control of a Tumor Suppressor PDCD4: Degradation Mechanisms of the Protein in Hepatocellular Carcinoma Cells. *Cell. Signal.* **2014**, *26*, 603–610, doi:10.1016/j.cellsig.2013.11.038.

146. Schmid, T.; Jansen, A.P.; Baker, A.R.; Hegamyer, G.; Hagan, J.P.; Colburn, N.H. Translation Inhibitor Pdcd4 Is Targeted for Degradation during Tumor Promotion. *Cancer Res* **2008**, *68*, 1254–1260, doi:10.1158/0008-5472.can-07-1719.

147. Kroczynska, B.; Sharma, B.; Eklund, E.A.; Fish, E.N.; Platanias, L.C. Regulatory Effects of Programmed Cell Death 4 (PDCD4) Protein in Interferon (IFN)-Stimulated Gene Expression and Generation of Type I IFN Responses. *Mol Cell Biol* **2012**, *32*, 2809–2822, doi:10.1128/mcb.00310-12.

148. Li, C.-W.; Lim, S.-O.; Xia, W.; Lee, H.-H.; Chan, L.-C.; Kuo, C.-W.; Khoo, K.-H.; Chang, S.-S.; Cha, J.-H.; Kim, T.; et al. Glycosylation and Stabilization of Programmed Death Ligand-1 Suppresses T-Cell Activity. *Nat. Commun.* **2016**, *7*, 12632, doi:10.1038/ncomms12632.

149. Zhou, M.; Kim, J.K.; Eng, G.W.L.; Forger, D.B.; Virshup, D.M. A Period2 Phosphoswitch Regulates and Temperature Compensates Circadian Period. *Mol Cell* **2015**, *60*, 77–88, doi:10.1016/j.molcel.2015.08.022.

150. Narasimamurthy, R.; Hunt, S.R.; Lu, Y.; Fustin, J.-M.; Okamura, H.; Partch, C.L.; Forger, D.B.; Kim, J.K.; Virshup, D.M. CK1δ/ε Protein Kinase Primes the PER2 Circadian Phosphoswitch. *Proc. Natl. Acad. Sci. United States Am.* **2018**, *115*, 5986–5991, doi:10.1073/pnas.1721076115.

151. Eide, E.J.; Woolf, M.F.; Kang, H.; Woolf, P.; Hurst, W.; Camacho, F.; Vielhaber, E.L.; Giovanni, A.; Virshup, D.M. Control of Mammalian Circadian Rhythm by CKIepsilon-Regulated Proteasome-Mediated PER2 Degradation. *Mol Cell Biol* **2005**, *25*, 2795–2807.

152. Masuda, S.; Narasimamurthy, R.; Yoshitane, H.; Kim, J.K.; Fukada, Y.; Virshup, D.M. Mutation of a PER2 Phosphodegron Perturbs the Circadian Phosphoswitch. *Proc. Natl. Acad. Sci.* **2020**, *117*, 10888–10896, doi:10.1073/pnas.2000266117.

153. Reischl, S.; Vanselow, K.; Westermark, P.O.; Thierfelder, N.; Maier, B.; Herzel, H.; Kramer, A. β-TrCP1-Mediated Degradation of PERIOD2 Is Essential for Circadian Dynamics. *J Biol Rhythm* **2007**, *22*, 375–386, doi:10.1177/0748730407303926.

154. Ohsaki, K.; Oishi, K.; Kozono, Y.; Nakayama, K.; Nakayama, K.I.; Ishida, N. The Role of {beta}-TrCP1 and {beta}-TrCP2 in Circadian Rhythm Generation by Mediating Degradation of Clock Protein PER2. *J Biochem* **2008**, *144*, 609–618, doi:10.1093/jb/mvn112.

155. Tatarskiy, V.V.; Simonov, Y.P.; Shcherbinin, D.S.; Brechalov, A.V.; Georgieva, S.G.; Soshnikova, N.V. Stability of the PHF10 Subunit of PBAF Signature Module Is Regulated by Phosphorylation: Role of β-TrCP. *Sci Rep-uk* **2017**, *7*, 5645, doi:10.1038/s41598-017-05944-3.

156. Li, X.; Liu, J.; Gao, T. β-TrCP-Mediated Ubiquitination and Degradation of PHLPP1 Are Negatively Regulated by Akt. *Mol Cell Biol* **2009**, *29*, 6192–6205, doi:10.1128/mcb.00681-09.

157. Gao, G.; Kun, T.; Sheng, Y.; Qian, M.; Kong, F.; Liu, X.; Yu, Z.; Zhang, H.; Zhang, Q.; Gu, J.; et al. SGT1 Regulates Akt Signaling by Promoting Beta-TrCP-Dependent PHLPP1 Degradation in Gastric Cancer Cells. *Mol Biol Rep* **2013**, *40*, 2947–2953, doi:10.1007/s11033-012-2363-8.

158. Liu, J.; Yuan, Y.; Xu, J.; Xiao, K.; Xu, Y.; Guo, T.; Zhang, L.; Wang, J.; Zheng, H. β-TrCP Restricts Lipopolysaccharide (LPS)-Induced Activation of TRAF6-IKK Pathway Upstream of IκBα Signaling. *Front Immunol* **2018**, *9*, 2930, doi:10.3389/fimmu.2018.02930.

159. Tian, Y.; Kolb, R.; Hong, J.-H.; Carroll, J.; Li, D.; You, J.; Bronson, R.; Yaffe, M.B.; Zhou, J.; Benjamin, T. TAZ Promotes PC2 Degradation through a SCF β-Trcp E3 Ligase Complex. *Mol Cell Biol* **2007**, *27*, 6383–6395, doi:10.1128/mcb.00254-07.

160. Kainulainen, M.; Lau, S.; Samuel, C.E.; Hornung, V.; Weber, F. NSs Virulence Factor of Rift Valley Fever Virus Engages the F-Box Proteins FBXW11 and β-TRCP1 To Degrade the Antiviral Protein Kinase PKR. *J Virol* **2016**, *90*, 6140–6147, doi:10.1128/jvi.00016-16.

161. Giráldez, S.; Galindo-Moreno, M.; Limón-Mortés, M.C.; Rivas, A.C.; Herrero-Ruiz, J.; Mora-Santos, M.; Sáez, C.; Japón, M.Á.; Tortolero, M.; Romero, F. G1/S Phase Progression Is Regulated by PLK1 Degradation through the CDK1/BTrCP Axis. *Faseb J* **2017**, *31*, 2925–2936, doi:10.1096/fj.201601108r.

162. Guderian, G.; Westendorf, J.; Uldschmid, A.; Nigg, E.A. Plk4 Trans-Autophosphorylation Regulates Centriole Number by Controlling BetaTrCP-Mediated Degradation. *J Cell Sci* **2010**, *123*, 2163–2169, doi:10.1242/jcs.068502.

163. Loveless, T.B.; Topacio, B.R.; Vashisht, A.A.; Galaang, S.; Ulrich, K.M.; Young, B.D.; Wohlschlegel, J.A.; Toczyski, D.P. DNA Damage Regulates Translation through β-TRCP Targeting of CReP. *PLoS Genet.* **2015**, *11*, e1005292, doi:10.1371/journal.pgen.1005292.

164. Coyaud, E.; Mis, M.; Laurent, E.M.N.; Dunham, W.H.; Couzens, A.L.; Robitaille, M.; Gingras, A.-C.; Angers, S.; Raught, B. BioID-Based Identification of Skp Cullin F-Box (SCF)β-TrCP1/2 E3 Ligase Substrates*[S]. *Mol. Cell. Proteom.* **2015**, *14*, 1781–1795, doi:10.1074/mcp.m114.045658.

165. Plotnikov, A.; Li, Y.; Tran, T.H.; Tang, W.; Palazzo, J.P.; Rui, H.; Fuchs, S.Y. Oncogene-Mediated Inhibition of Glycogen Synthase Kinase 3β Impairs Degradation of Prolactin Receptor. *Cancer Res* **2008**, *68*, 1354–1361, doi:10.1158/0008-5472.can-07-6094.

166. Li, Y.; Kumar, K.G.S.; Tang, W.; Spiegelman, V.S.; Fuchs, S.Y. Negative Regulation of Prolactin Receptor Stability and Signaling Mediated by SCF β-TrCP E3 Ubiquitin Ligase. *Mol Cell Biol* **2004**, *24*, 4038–4048, doi:10.1128/mcb.24.9.4038-4048.2004.

167. Shen, S.-M.; Zhang, C.; Ge, M.-K.; Dong, S.-S.; Xia, L.; He, P.; Zhang, N.; Ji, Y.; Yang, S.; Yu, Y.; et al. PTENα and PTENβ Promote Carcinogenesis through WDR5 and H3K4 Trimethylation. *Nat. Cell Biol.* **2019**, *21*, 1436–1448, doi:10.1038/s41556-019-0409-z.

168. Wang, D.; Zhang, P.; Gao, K.; Tang, Y.; Jin, X.; Zhang, Y.; Yi, Q.; Wang, C.; Yu, L. PLK1 and β-TrCP-Dependent Ubiquitination and Degradation of Rap1GAP Controls Cell Proliferation. *Plos One* **2014**, *9*, e110296, doi:10.1371/journal.pone.0110296.

169. Zhou, X.; Li, T.-T.; Feng, X.; Hsiang, E.; Xiong, Y.; Guan, K.-L.; Lei, Q.-Y. Targeted Polyubiquitylation of RASSF1C by the Mule and SCFβ-TrCP Ligases in Response to DNA Damage. *Biochem. J.* **2011**, *441*, 227–236, doi:10.1042/bj20111500.

170. Guardavaccaro, D.; Frescas, D.; Dorrello, N.V.; Peschiaroli, A.; Multani, A.S.; Cardozo, T.; Lasorella, A.; Iavarone, A.; Chang, S.; Hernando, E.; et al. Control of Chromosome Stability by the β-TrCP–REST–Mad2 Axis. *Nature* **2008**, *452*, 365–369, doi:10.1038/nature06641.

171. Nesti, E.; Corson, G.M.; McCleskey, M.; Oyer, J.A.; Mandel, G. C-Terminal Domain Small Phosphatase 1 and MAP Kinase Reciprocally Control REST Stability and Neuronal Differentiation. *Proc National Acad Sci* **2014**, *111*, E3929–E3936, doi:10.1073/pnas.1414770111.

172. Kaneko, N.; Hwang, J.-Y.; Gertner, M.; Pontarelli, F.; Zukin, R.S. Casein Kinase 1 Suppresses Activation of REST in Insulted Hippocampal Neurons and Halts Ischemia-Induced Neuronal Death. *J. Neurosci.* **2014**, *34*, 6030–6039, doi:10.1523/jneurosci.4045-13.2014.

173. Tanghe, G.; Urwyler-Rösselet, C.; Groote, P.D.; Dejardin, E.; Bock, P.-J.D.; Gevaert, K.; Vandenabeele, P.; Declercq, W. RIPK4 Activity in Keratinocytes Is Controlled by the SCFβ-TrCP Ubiquitin Ligase to Maintain Cortical Actin Organization. *Cell. Mol. Life Sci.* **2018**, *75*, 2827–2841, doi:10.1007/s00018-018-2763-6.

174. Fukuchi, M.; Imamura, T.; Chiba, T.; Ebisawa, T.; Kawabata, M.; Tanaka, K.; Miyazono, K. Ligand-Dependent Degradation of Smad3 by a Ubiquitin Ligase Complex of ROC1 and Associated Proteins. *Mol Biol Cell* **2001**, *12*, 1431–1443, doi:10.1091/mbc.12.5.1431.

175. Demagny, H.; Araki, T.; De Robertis, E.M. The Tumor Suppressor Smad4/DPC4 Is Regulated by Phosphorylations That Integrate FGF, Wnt, and TGF-β Signaling. *Cell Rep.* **2014**, *9*, 688–700, doi:10.1016/j.celrep.2014.09.020.

176. Wan, M.; Tang, Y.; Tytler, E.M.; Lu, C.; Jin, B.; Vickers, S.M.; Yang, L.; Shi, X.; Cao, X. Smad4 Protein Stability Is Regulated by Ubiquitin Ligase SCFβ-TrCP1. *J Biol Chem* **2004**, *279*, 14484–14487, doi:10.1074/jbc.c400005200.

177. Xu, Y.; Lee, S.-H.; Kim, H.S.; Kim, N.H.; Piao, S.; Park, S.-H.; Jung, Y.S.; Yook, J.I.; Park, B.-J.; Ha, N.-C. Role of CK1 in GSK3β-Mediated Phosphorylation and Degradation of Snail. *Oncogene* **2010**, *29*, 3124–3133, doi:10.1038/onc.2010.77.

178. Zhou, B.P.; Deng, J.; Xia, W.; Xu, J.; Li, Y.M.; Gunduz, M.; Hung, M.-C. Dual Regulation of Snail by GSK-3β-Mediated Phosphorylation in Control of Epithelial–Mesenchymal Transition. *Nat Cell Biol* **2004**, *6*, 931–940, doi:10.1038/ncb1173.

179. Yook, J.I.; Li, X.-Y.; Ota, I.; Fearon, E.R.; Weiss, S.J. Wnt-Dependent Regulation of the E-Cadherin Repressor Snail*. *J Biol Chem* **2005**, *280*, 11740–11748, doi:10.1074/jbc.m413878200.

180. Wei, S.; Chuang, H.-C.; Tsai, W.-C.; Yang, H.-C.; Ho, S.-R.; Paterson, A.J.; Kulp, S.K.; Chen, C.-S. Thiazolidinediones Mimic Glucose Starvation in Facilitating Sp1 Degradation through the Up-Regulation of β-Transducin Repeat-Containing Protein. *Mol Pharmacol* **2009**, *76*, 47–57, doi:10.1124/mol.109.055376.

181. Chen, Y.-J.; Liu, W.-H.; Chang, L.-S. Hydroquinone-Induced FOXP3-ADAM17-Lyn-Akt-P21 Signaling Axis Promotes Malignant Progression of Human Leukemia U937 Cells. *Arch. Toxicol.* **2017**, *91*, 983–997, doi:10.1007/s00204-016-1753-4.

182. Huang, C.-H.; Lee, Y.-C.; Chen, Y.-J.; Wang, L.-J.; Shi, Y.-J.; Chang, L.-S. Quinacrine Induces the Apoptosis of Human Leukemia U937 Cells through FOXP3/MiR-183/β-TrCP/SP1 Axis-Mediated BAX Upregulation. *Toxicol Appl Pharm* **2017**, *334*, 35–46, doi:10.1016/j.taap.2017.08.019.

183. Senese, S.; Cheung, K.; Lo, Y.-C.; Gholkar, A.A.; Xia, X.; Wohlschlegel, J.A.; Torres, J.Z. A Unique Insertion in STARD9's Motor Domain Regulates Its Stability. *Mol. Biol. Cell* **2015**, *26*, 440–452, doi:10.1091/mbc.e14-03-0829.

184. Arquint, C.; Cubizolles, F.; Morand, A.; Schmidt, A.; Nigg, E.A. The SKP1-Cullin-F-Box E3 Ligase BTrCP and CDK2 Cooperate to Control STIL Abundance and Centriole Number. *Open Biol.* **2018**, *8*, 170253, doi:10.1098/rsob.170253.

185. Krshnan, L.; Siu, W.S.; Weijer, M.V. de; Hayward, D.; Guerrero, E.N.; Gruneberg, U.; Carvalho, P. Regulated Degradation of the Inner Nuclear Membrane Protein SUN2 Maintains Nuclear Envelope Architecture and Function. *eLife* **2022**, *11*, doi:10.7554/elife.81573.

186. Liu, C.-Y.; Zha, Z.-Y.; Zhou, X.; Zhang, H.; Huang, W.; Zhao, D.; Li, T.; Chan, S.W.; Lim, C.J.; Hong, W.; et al. The Hippo Tumor Pathway Promotes TAZ Degradation by Phosphorylating a Phosphodegron and Recruiting the SCFβ-TrCP E3 Ligase*. *J. Biological Chem.* **2010**, *285*, 37159–37169, doi:10.1074/jbc.m110.152942.

187. Huang, W.; Lv, X.; Liu, C.; Zha, Z.; Zhang, H.; Jiang, Y.; Xiong, Y.; Lei, Q.-Y.; Guan, K.-L. The N-Terminal Phosphodegron Targets TAZ/WWTR1 Protein for SCFβ-TrCP-Dependent Degradation in Response to Phosphatidylinositol 3-Kinase Inhibition*. *J Biol Chem* **2012**, *287*, 26245–26253, doi:10.1074/jbc.m112.382036.

188. Zhang, Y.; Ding, H.; Wang, X.; Wang, X.; Wan, S.; Xu, A.; Gan, R.; Ye, S.-D. MK2 Promotes Tfcp2l1 Degradation via β-TrCP Ubiquitin Ligase to Regulate Mouse Embryonic Stem Cell Self-Renewal. *Cell Reports* **2021**, *37*, 109949, doi:10.1016/j.celrep.2021.109949.

189. Zhu, G.; Fan, Z.; Ding, M.; Mu, L.; Liang, J.; Ding, Y.; Fu, Y.; Huang, B.; Wu, W. DNA Damage Induces the Accumulation of Tiam1 by Blocking β-TrCP-Dependent Degradation*. *J Biol Chem* **2014**, *289*, 15482–15494, doi:10.1074/jbc.m114.553388.

190. Magliozzi, R.; Kim, J.; Low, T.Y.; Heck, A.J.R.; Guardavaccaro, D. Degradation of Tiam1 by Casein Kinase 1 and the SCFβTrCP Ubiquitin Ligase Controls the Duration of MTOR-S6K Signaling*. *J. Biol. Chem.* **2014**, *289*, 27400–27409, doi:10.1074/jbc.m114.575571.

191. Lou, Y.; Han, M.; Song, Y.; Zhong, J.; Zhang, W.; Chen, Y.H.; Wang, H. The SCFβ-TrCP E3 Ubiquitin Ligase Regulates Immune Receptor Signaling by Targeting the Negative Regulatory Protein TIPE2. *J. Immunol.* **2020**, *204*, 2122–2132, doi:10.4049/jimmunol.1901142.

192. Shu, J.; Cui, D.; Ma, Y.; Xiong, X.; Sun, Y.; Zhao, Y. SCFβ-TrCP-Mediated Degradation of TOP2β Promotes Cancer Cell Survival in Response to Chemotherapeutic Drugs Targeting Topoisomerase II. *Oncogenesis* **2020**, *9*, 8, doi:10.1038/s41389-020-0196-1.

193. Xia, Y.; Padre, R.C.; Mendoza, T.H.D.; Bottero, V.; Tergaonkar, V.B.; Verma, I.M. Phosphorylation of P53 by IκB Kinase 2 Promotes Its Degradation by β-TrCP. *Proc National Acad Sci* **2009**, *106*, 2629–2634, doi:10.1073/pnas.0812256106.

194. Krishnamurthy, P.M.; Shukla, S.; Ray, P.; Mehra, R.; Nyati, M.K.; Lawrence, T.S.; Ray, D. Involvement of P38-BTrCP-Tristetraprolin-TNFα Axis in Radiation Pneumonitis. *Oncotarget* **2017**, *8*, 47767–47779, doi:10.18632/oncotarget.17770.

195. Shi, P.; Zhu, S.; Lin, Y.; Liu, Y.; Liu, Y.; Chen, Z.; Shi, Y.; Qian, Y. Persistent Stimulation with Interleukin-17 Desensitizes Cells Through SCFβ-TrCP-Mediated Degradation of Act1. *Sci. Signal.* **2011**, *4*, ra73, doi:10.1126/scisignal.2001653.

196. Wang, C.; Xiao, H.; Ma, J.; Zhu, Y.; Yu, J.; Sun, L.; Sun, H.; Liu, Y.; Jin, C.; Huang, H. The F-Box Protein β-TrCP Promotes Ubiquitination of TRF1 and Regulates the ALT-Associated PML Bodies Formation in U2OS Cells. *Biochem. Biophys. Res. Commun.* **2013**, *434*, 728–734, doi:10.1016/j.bbrc.2013.03.096.

197. Qiao, Y.; Zhang, Y.; Wang, J. Ubiquitin E3 Ligase SCFβ-TRCP Regulates TRIB2 Stability in Liver Cancer Cells. *Biochem. Biophys. Res. Commun.* **2013**, *441*, 555–559, doi:10.1016/j.bbrc.2013.10.123.

198. Li, C.-W.; Xia, W.; Lim, S.-O.; Hsu, J.L.; Huo, L.; Wu, Y.; Li, L.-Y.; Lai, C.-C.; Chang, S.-S.; Hsu, Y.-H.; et al. AKT1 Inhibits Epithelial-to-Mesenchymal Transition in Breast Cancer through Phosphorylation-Dependent Twist1 Degradation. *Cancer Res* **2016**, *76*, 1451–1462, doi:10.1158/0008-5472.can-15-1941.

199. Zhong, J.; Ogura, K.; Wang, Z.; Inuzuka, H. Degradation of the Transcription Factor Twist, an Oncoprotein That Promotes Cancer Metastasis. *Discov. Med.* **2013**, *15*, 7–15.

200. Chen, H.; Ma, H.; Inuzuka, H.; Diao, J.; Lan, F.; Shi, Y.G.; Wei, W.; Shi, Y. DNA Damage Regulates UHRF1 Stability via the SCFβ-TrCP E3 Ligase. *Mol. Cell. Biol.* **2013**, *33*, 1139–1148, doi:10.1128/mcb.01191-12.

201. Deng, R.; Zhang, H.-L.; Huang, J.-H.; Cai, R.-Z.; Wang, Y.; Chen, Y.-H.; Hu, B.-X.; Ye, Z.-P.; Li, Z.-L.; Mai, J.; et al. MAPK1/3 Kinase-Dependent ULK1 Degradation Attenuates Mitophagy and Promotes Breast Cancer Bone Metastasis. *Autophagy* **2021**, *17*, 3011–3029, doi:10.1080/15548627.2020.1850609.

202. Shaik, S.; Nucera, C.; Inuzuka, H.; Gao, D.; Garnaas, M.; Frechette, G.; Harris, L.; Wan, L.; Fukushima, H.; Husain, A.; et al. SCF(β-TRCP) Suppresses Angiogenesis and Thyroid Cancer Cell Migration by Promoting Ubiquitination and Destruction of VEGF Receptor 2. *J Exp Medicine* **2012**, *209*, 1289–1307, doi:10.1084/jem.20112446.

203. WU, W.; ZHANG, D.; PAN, D.; ZUO, G.; REN, X.; CHEN, S. Downregulation of Vascular Endothelial Growth Factor Receptor-2 under Oxidative Stress Conditions Is Mediated by β-Transduction Repeat-Containing Protein via Glycogen Synthase Kinase-3β Signaling. *Int. J. Mol. Med.* **2016**, *37*, 911–920, doi:10.3892/ijmm.2016.2493.

204. Meyer, R.D.; Srinivasan, S.; Singh, A.J.; Mahoney, J.E.; Gharahassanlou, K.R.; Rahimi, N. PEST Motif Serine and Tyrosine Phosphorylation Controls Vascular Endothelial Growth Factor Receptor 2 Stability and Downregulation. *Mol Cell Biol* **2011**, *31*, 2010–2025, doi:10.1128/mcb.01006-10.

205. Watanabe, N.; Arai, H.; Nishihara, Y.; Taniguchi, M.; Watanabe, N.; Hunter, T.; Osada, H. M-Phase Kinases Induce Phospho-Dependent Ubiquitination of Somatic Wee1 by SCFbeta-TrCP. *P Natl Acad Sci Usa* **2004**, *101*, 4419–4424.

206. Watanabe, N.; Arai, H.; Iwasaki, J.; Shiina, M.; Ogata, K.; Hunter, T.; Osada, H. Cyclin-Dependent Kinase (CDK) Phosphorylation Destabilizes Somatic Wee1 via Multiple Pathways. *P Natl Acad Sci Usa* **2005**, *102*, 11663–11668, doi:10.1073/pnas.0500410102.

207. Yousafzai, N.A.; Zhou, Q.; Xu, W.; Shi, Q.; Xu, J.; Feng, L.; Chen, H.; Shin, V.Y.; Jin, H.; Wang, X. SIRT1 Deacetylated and Stabilized XRCC1 to Promote Chemoresistance in Lung Cancer. *Cell Death Dis* **2019**, *10*, 363, doi:10.1038/s41419-019-1592-3.

208. Zhao, B.; Li, L.; Tumaneng, K.; Wang, C.-Y.; Guan, K.-L. A Coordinated Phosphorylation by Lats and CK1 Regulates YAP Stability through SCFβ-TRCP. *Gene Dev* **2010**, *24*, 72–85, doi:10.1101/gad.1843810.

209. Guo, Q.; Quan, M.; Dong, J.; Bai, J.; Wang, J.; Han, R.; Wang, W.; Cai, Y.; Lv, Y.-Q.; Chen, Q.; et al. The WW Domains Dictate Isoform-Specific Regulation of YAP1 Stability and Pancreatic Cancer Cell Malignancy. *Theranostics* **2020**, *10*, 4422–4436, doi:10.7150/thno.42795.

210. Zhu, Y.; Zhou, Q.; Zhu, G.; Xing, Y.; Li, S.; Ren, N.; Liu, T.; Zhu, A.; Bai, Y.; Piao, D. GSK-3β Phosphorylation-Dependent Degradation of ZNF281 by β-TrCP2 Suppresses Colorectal Cancer Progression. *Oncotarget* **2017**, *8*, 88599–88612, doi:10.18632/oncotarget.20100.

211. Ci, Y.; Li, X.; Chen, M.; Zhong, J.; North, B.J.; Inuzuka, H.; He, X.; Li, Y.; Guo, J.; Dai, X. SCFβ-TRCP E3 Ubiquitin Ligase Targets the Tumor Suppressor ZNRF3 for Ubiquitination and Degradation. *Protein Cell* **2018**, *9*, 879–889, doi:10.1007/s13238-018-0510-2.
